# A Pan-Cancer Multi-Omics Analysis of CAD: Integrating CRISPR and Metabolomics Data to Unravel the Metabolic–Immune Axis and Immunotherapy Response

**DOI:** 10.3390/biomedicines14061218

**Published:** 2026-05-28

**Authors:** Yiyan Li, Aoxue Xing, Wenzheng Li, Yiman Zhang, Kejuan Zhang, Tianhao Xie, Gang Wu, Wei Zhang

**Affiliations:** 1 Department of Gastrointestinal Surgery, People’s Hospital of Zhengzhou University, Henan Provincial People’s Hospital, Henan Provincial People’s Clinical Medical School of Zhengzhou University, Henan Provincial Research Center for Precision Control Engineering of Digestive Tract Tumors, Zhengzhou 450003, China; lyy3010@163.com (Y.L.);; 2 Department of Gastrointestinal Surgery, People’s Hospital of Henan University, Henan Provincial People’s Hospital, Henan Provincial People’s Clinical Medical School of Henan University, Henan Provincial Research Center for Precision Control Engineering of Digestive Tract Tumors, Zhengzhou 450003, China

**Keywords:** CAD, pan-cancer analysis, immunotherapy, prognosis, pyrimidine metabolism, tumor microenvironment, biomarker, meta-analysis

## Abstract

**Background:** CAD (Carbamoyl-Phosphate Synthetase 2, Aspartate Transcarbamylase, and Dihydroorotase) is a pivotal tri-functional enzyme complex associated with the rate-limiting steps of the de novo pyrimidine biosynthetic pathway. Despite its established metabolic role, the multi-dimensional involvement of CAD in the pan-cancer landscape—specifically regarding its regulation of the metabolic–immune axis and its impact on immunotherapy response—remains to be fully elucidated. **Methods:** We performed a systematic pan-cancer multi-omics analysis integrating TCGA, GTEx, and DepMap datasets to evaluate CAD expression, genomic alterations, and diagnostic potential. In addition, multiple immunotherapy cohorts were integrated for meta-analysis, and metabolomic data were incorporated to explore CAD-associated metabolic features. **Results:** CAD was significantly upregulated in 17 cancer types, with protein-level evidence supporting this trend. CAD also showed high diagnostic accuracy in several malignancies, particularly LAML and CHOL (AUC approximately 1.0). In immunotherapy-related analyses, CAD expression was positively associated with TMB, MSI, and initial therapeutic response, but was also linked to worse long-term overall survival in pooled cohorts (HR = 1.42, 95% CI: 1.19–1.70). Integrative metabolomic analyses further suggested that high CAD expression was associated with pyrimidine metabolite accumulation and altered amino acid metabolism, indicating a potential link between CAD-related metabolic reprogramming and the tumor immune microenvironment. **Conclusions:** CAD may represent a promising candidate biomarker across multiple malignancies. Notably, its association with immunotherapy-related features, together with the observed discordance between response-associated signals and long-term survival, warrants further mechanistic and clinical validation.

## 1. Introduction

Despite significant advances in the diagnosis and treatment of cancer, cancer remains a serious global health challenge and one of the leading causes of death in humans [1], While current clinical markers encompass diverse dimensions—such as genomic variants (e.g., BRCA1/2), proteomic signatures (e.g., Caveolin-1), and liquid biopsies (e.g., CTCs and ctDNA)—the pronounced molecular heterogeneity of tumors underscores the urgent need for robust, multi-dimensional biomarkers that can concurrently facilitate early detection, accurate prognostic assessment, and effective treatment response monitoring, particularly in the era of precision medicine and immunotherapy [2,3,4,5].

The CAD gene encodes a multidomain protein [6] that catalyzes the first three rate-limiting enzymatic steps within the biosynthetic framework of de novo pyrimidine generation [7]. Through the synergistic action of its enzymatic domains, CAD facilitates the conversion of glutamine, ATP, and bicarbonate to *N*-carbamyl-L-aspartate, a key step in pyrimidine ring formation [8]. Pyrimidines are essential precursors for DNA and RNA synthesis [9], and CAD proteins ensure a supply of nucleic acids to support cell growth, proliferation, metabolism, and other biological functions [10]. A fundamental feature of malignant progression is metabolic reprogramming, which supports the elevated biosynthetic demands of aggressive tumor cells [11] and influences the immune microenvironment within tumors, ultimately affecting immunotherapy response and long-term patient prognosis [12]. As a nucleotide metabolism enzyme, CAD has been implicated in cancer-related metabolic network imbalances in previous studies [13]. For instance, the downregulation of urea cycle enzymes (e.g., carbamoyl-phosphate synthetase 1 (CPS1) and argininosuccinate synthase (ASS1)) can divert nitrogenous flux and glutamine resources toward the CAD complex [14,15]. This metabolic rewiring accelerates pyrimidine output, providing essential raw materials to drive the proliferation and invasion of malignant cells [16].

However, current research on CAD is largely confined to its oncogenic roles in specific malignancies, such as hepatocellular carcinoma and glioblastoma. Its broader significance as a clinical biomarker across the pan-cancer spectrum—and specifically how CAD-mediated pyrimidine synthesis coordinates with the tumor immune microenvironment to influence immunotherapy efficacy—remains to be systematically investigated.

To address these identified gaps, the present study systematically integrates CRISPR-Cas9 dependency and metabolomics datasets to comprehensively characterize the functional landscape of CAD. Furthermore, by incorporating 53 independent immunotherapy cohorts, we performed a systematic meta-analysis to evaluate the association between CAD expression and patient prognosis. Ultimately, this study provides a comprehensive computational framework that identifies CAD as a promising candidate biomarker linking metabolic reprogramming to tumor immunity, warranting future experimental and prospective clinical validation.

## 2. Materials and Methods

### 2.1. CAD Expression Analysis

In this study, CAD mRNA expression was evaluated across multiple public databases. Physiological expression patterns of CAD in normal human tissues were first assessed using the Human Protein Atlas (HPA; https://www.proteinatlas.org/), from which normalized transcripts per million (nTPM) values were obtained to facilitate cross-tissue comparison [17]. Subsequently, we utilized the GEPIA2 (Interactive Analysis of Gene Expression 2, http://gepia2.cancer-pku.cn/) platform [18], which integrates data from the TCGA and GTEx projects [19], to compare CAD expression between 33 types of tumor tissues and their corresponding normal controls. Differential expression analysis was performed using the GEPIA2 standard pipeline with a threshold of |log2 fold change| > 1 and a q-value < 0.01 based on one-way ANOVA. Expression distributions were visualized as box plots using log2(TPM + 1)-transformed data.

### 2.2. Pan-Cancer Diagnostic Value Analysis

To evaluate the diagnostic performance of CAD in distinguishing tumor tissues from normal tissues, receiver operating characteristic (ROC) analysis was performed using a standardized pan-cancer cohort obtained from the UCSC Xena database (https://xenabrowser.net/) [20]. The integrated dataset included samples from TCGA, TARGET, and GTEx, providing a unified resource for malignant and normal tissue comparisons. Samples lacking CAD expression data or tissue-type annotations were excluded from the analysis. CAD expression values were transformed as log2(x + 0.001) prior to analysis. The area under the ROC curve (AUC) was calculated for each cancer type using the pROC package (v1.18.0) in R software (v4.5.0) [21,22]. An AUC > 0.7 was considered indicative of good diagnostic performance, whereas an AUC > 0.9 was considered indicative of excellent diagnostic potential [23]. The AUC values across cancer types were visualized using bar plots generated with the ggplot2 package.

### 2.3. Genetic Alteration Analysis

To characterize the genomic alteration landscape of CAD, data were retrieved from cBioPortal for Cancer Genomics (v5.4.1; https://www.cbioportal.org/) [24]. Mutation frequency, mutation type, mutation site, and copy number alteration profiles of CAD across TCGA cohorts were obtained from the corresponding cBioPortal modules. Complementary analyses of CAD mutation frequency and mutation patterns across tumor types were performed using TIMER2.0 [25] and SangerBox (http://sangerbox.com/ accessed on 25 December 2025) [26]. The association between CAD mRNA expression and copy number alterations was evaluated using Spearman’s rank correlation analysis in SangerBox. In addition, the relationship between CAD expression and somatic mutation patterns was further explored using the “Mutational Landscape” module of the CAMOIP platform [27].

### 2.4. Survival Analysis

For survival analysis, we followed the standard SangerBox protocols to generate forest plots for Overall Survival (OS) and Disease-Free Survival (DFS) across TCGA datasets. Additionally, we produced Kaplan–Meier curves and survival maps using the “Survival Analysis” and “Survival Map” tools within GEPIA2.0 [18]. For these analyses, patients were divided into high- and low-expression groups based on a median cutoff (50% threshold).

To further assess whether CAD was independently associated with prognosis, multivariable Cox proportional hazards regression models were constructed using the TIMER2.0 survival module. We included all 33 TCGA cancer types in our initial screening; however, multivariable analysis was performed only in cohorts where the necessary clinical covariates (age, gender, race, pathological stage, and tumor purity) were adequately represented and where the cohort size and number of outcome events were sufficient to ensure model stability (i.e., avoiding overfitting or unreliable coefficient estimates). For each model, complete-case analysis was applied, retaining only cases with non-missing values for all included variables. Detailed lists of the cancer types that successfully underwent multivariable analysis and their respective model statistics are provided in Supplementary Table S2.

### 2.5. Metabolomics and Gene Dependency Analysis

To investigate the metabolic features and gene dependency associated with CAD in a pan-cancer context, multi-omics data were obtained from the Cancer Dependency Map (DepMap) portal (https://depmap.org/portal/ accessed on 25 December 2025) [28]. Three datasets were included in this analysis: cancer cell line transcriptomic profiles (CCLE_expression.csv), genome-wide CRISPR knockout screening data (CRISPR_gene_effect.csv) to quantify CAD-related dependency, and metabolomic profiles (CCLE_metabolomics.csv), which represent the relative abundances of polar and semi-polar metabolites measured by liquid chromatography–mass spectrometry (LC–MS) [29]. During preprocessing, only cell lines with complete data available across all three datasets were retained, resulting in 224 cancer cell lines for downstream analysis. Only metabolic features annotated as pure metabolites were included, whereas ambiguous features were excluded. Spearman’s rank correlation analysis was performed to evaluate the associations of metabolite abundance with CAD mRNA expression and CAD CRISPR dependency scores. To control for multiple testing in this high-dimensional analysis, *p*-values were adjusted using the Benjamini–Hochberg method, and a false discovery rate (FDR) < 0.05 was considered statistically significant. The most significantly associated metabolites were visualized using volcano plots and bar charts generated with the ggplot2 package.

### 2.6. Correlation of CAD with Cancer Stemness and RNA Modification

The association of CAD with tumor stemness and RNA epigenetic regulation was systematically evaluated. Cancer stemness indices, including mRNA-based stemness scores (RNAss and EREG.EXPss) and DNA methylation-based stemness scores (DNAss, EREG.METHss, DMPss, and ENHss), were obtained from the SangerBox platform [30]. Pearson correlation analysis was performed to assess the relationships between CAD expression and these stemness indices, and the results were visualized using R software. In addition, the associations between CAD expression and key regulators of major RNA modifications, including m6A, m5C, and m1A regulators, were analyzed. These RNA modification regulators were classified into three functional categories: writers, erasers, and readers [31]. Correlation analysis and visualization for RNA modification regulators were conducted using the SangerBox platform. For all analyses, Pearson’s correlation coefficient was used to estimate the strength of association, and *p* < 0.05 was considered statistically significant.

### 2.7. CAD Co-Expression Gene Analysis

CAD co-expressed genes and their corresponding Pearson correlation coefficients were downloaded from the “Co-expression” section of cBioPortal. Genes positively correlated with CAD with a Pearson correlation coefficient r > 0.3 were screened. The package clusterprofiler ang ggplot2 in R software was used to perform and visualize GO (Gene Ontology) term enrichment analysis and KEGG (Kyoto Encyclopedia of Genes and Genomes) enrichment analysis [32,33].

### 2.8. Assessment of CAD in Tumor Immune Landscape Evaluation and Clinical Benefit of Immunotherapy

The immunological relevance of CAD was evaluated using the SangerBox platform. Pearson correlation analysis was performed to assess the associations between CAD expression and immune-related scores, including ESTIMATE score, Stromal score, and Immune score [34]. The infiltration levels of six major tumor-infiltrating immune cell types, including macrophages, neutrophils, dendritic cells, B cells, CD4+ T cells, and CD8+ T cells, were also estimated. In addition, the correlations between CAD expression and key immunogenicity-related biomarkers, including tumor mutational burden (TMB), microsatellite instability (MSI), and neoantigen counts (NEO), were analyzed across TCGA cohorts. The co-expression patterns of CAD with immunomodulatory genes, including both immunostimulatory and immunosuppressive factors, were further investigated. Correlation results were visualized using radar plots and heatmaps generated in R software (version 4.5.0).

The relationship between CAD expression and response to immune checkpoint blockade (ICB) therapy was first evaluated using the ROC Plotter platform [35]. In the immunotherapy module, all available treatment categories were included, including anti-PD-1 monotherapy, anti-PD-L1 monotherapy, and combination regimens, and only pre-treatment biopsy samples were retained for analysis. Patients were stratified into high- and low-expression groups according to the median CAD expression level to assess the association between CAD expression and treatment response.

To further validate the predictive value of CAD in immunotherapy, a meta-analysis was conducted using 53 independent immunotherapy cohorts obtained from the Cancer Immunology Data Engine (CIDE) [36]. Eligible cohorts were required to contain bulk RNA-seq data and matched clinical survival information following ICB treatment, with biopsy samples restricted to the pre-treatment setting. These cohorts mainly included melanoma, non-small cell lung cancer, urothelial carcinoma, and renal cell carcinoma. Hazard ratios (HRs) and corresponding 95% CIs were collected for each cohort. A random-effects model was applied to estimate the pooled HR and 95% CI to account for inter-cohort heterogeneity [37]. Heterogeneity was assessed using Cochran’s *Q* test and the *I*^2^ statistic. Sensitivity analysis was performed using a leave-one-out approach to examine the robustness of the pooled results. Potential publication bias was evaluated using Egger’s test. Meta-analysis results were visualized as forest plots in R software (version 4.5.0).

### 2.9. Data Preprocessing

To minimize batch effects between the TCGA and GTEx datasets, we utilized the harmonized RNA-seq data from the UCSC Xena Toil recompute project. In this project, raw sequence data from both TCGA and GTEx were reprocessed using a uniform bioinformatic pipeline (STAR-Salmon), which eliminates technical variability caused by different computational methods. For gene expression analysis, Transcripts Per Million (TPM) values were used and log2(TPM + 1) transformed for normalization. For clinical correlation and survival analysis, patients with a recorded survival time of 0 days or missing critical clinical information (such as pathological stage) were excluded to ensure data integrity. Sample quality control followed the stringent standards set by the TCGA and GTEx consortiums, and only primary tumor and normal tissue samples were retained for this study.

### 2.10. Statistical Analysis and Visualization

Statistical analyses were conducted using a combination of public web platforms and R software (version 4.5.0). Data visualization and supplementary statistical analyses were performed in R using packages including ggplot2 (v3.5.1), pheatmap (v1.0.12), pROC (v1.18.0), forestploter (v1.1.1), clusterprofiler (v.4.16.0), and meta (v7.0-0). Pearson correlation analysis was primarily used for transcriptome-based association analyses, such as correlations with stemness indices, RNA modification regulators, and immune-related scores. Spearman’s rank correlation analysis was applied to analyses involving non-normally distributed data, including metabolomics, CRISPR dependency, and copy number alteration-related analyses.

For survival analysis, multivariable Cox proportional hazards models were obtained using the TIMER2.0 survival module. CAD expression was analyzed together with available clinical covariates, including age, gender, race, pathological stage, and tumor purity, depending on data availability in each cancer type. Only cases with complete values for all variables included in a given model were retained. Hazard ratios (HRs), 95% confidence intervals (CIs), and corresponding *p* values were extracted from each model.

For the meta-analysis of immunotherapy cohorts, effect sizes were synthesized using hazard ratios and corresponding 95% CIs under a random-effects model. Inter-study heterogeneity was assessed using Cochran’s *Q* test and the *I*^2^ statistic. Sensitivity analysis was performed using a leave-one-out approach, and potential publication bias was evaluated using Egger’s test.

For metabolomics association analyses involving multiple simultaneous tests, *p* values were adjusted using the Benjamini–Hochberg false discovery rate (FDR) method, and FDR < 0.05 was considered statistically significant. For analyses performed directly on public platforms, statistical significance was determined according to the default or explicitly specified criteria of each platform. Significance levels in figures were denoted as follows: * *p* < 0.05; ** *p* < 0.01; *** *p* < 0.001.

## 3. Results

### 3.1. CAD Expression Is Upregulated in Multiple Cancers and Associated with Adverse Clinicopathological Features

**Figure 1:**
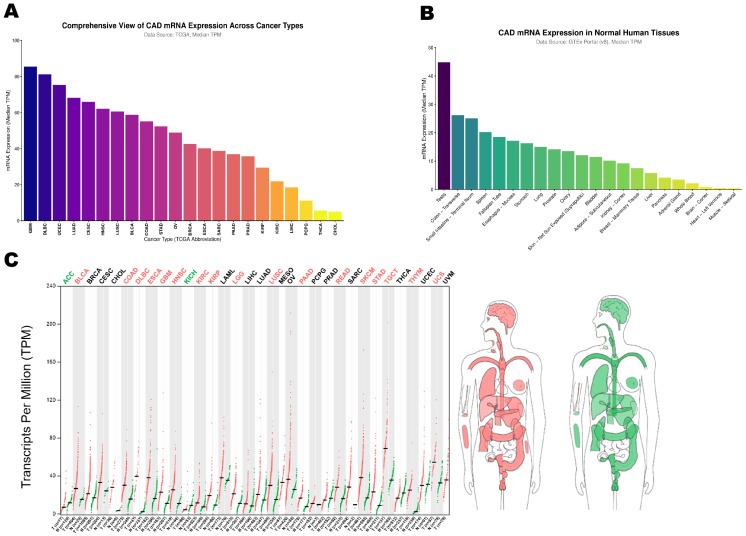
Pan-cancer expression landscape of CAD. (**A**) Comprehensive view of CAD mRNA expression levels (median TPM) across diverse cancer types derived from the TCGA database. (**B**) Baseline CAD mRNA expression levels (median TPM) in normal human tissues derived from the GTEx Portal (v8). (**C**) Differential expression analysis of CAD between tumor tissues and normal healthy tissues across multiple cancer cohorts. The scatter plot (**left**) details the distribution of transcripts per million (TPM) values, while the anatomical human body diagram (**right**) visually maps the organs exhibiting dysregulated expression. Color coding: In panel (**C**), red indicates tumor tissues (T) or significant upregulation, whereas green indicates normal tissues (N) or significant downregulation. The black dash indicates the median expression level for each group. Abbreviations: TCGA, The Cancer Genome Atlas; GTEx, Genotype-Tissue Expression; TPM, Transcripts Per Million. A comprehensive list mapping all cancer type abbreviations to their full clinical names is provided in Supplementary Table S1. Statistical analysis: Differential expression between groups was evaluated using the Wilcoxon rank-sum test. For multi-group comparisons (e.g., clinical stages), the Kruskal–Wallis test was employed.

Previous studies have reported aberrant CAD expression in several malignancies, including hepatocellular carcinoma, colorectal cancer, and glioma [38,39,40]. To systematically characterize the expression pattern of CAD across normal tissues and human cancers, we analyzed publicly available GTEx and TCGA datasets. In normal tissues, CAD mRNA expression displayed marked tissue specificity, with the highest levels observed in the testis, followed by the transverse colon and terminal ileum, whereas relatively low expression was detected in terminally differentiated tissues such as skeletal muscle and the left ventricle of the heart (Figure 1A).

We next examined CAD expression across 33 tumor types in TCGA (Figure 1B). Among them, glioblastoma multiforme (GBM), diffuse large B-cell lymphoma (DLBC), and uterine corpus endometrial carcinoma (UCEC) showed the highest CAD expression levels. Tumor-normal comparison using the GEPIA2 platform further showed that CAD was significantly upregulated in 17 of 33 tumor types (Figure 1C), whereas decreased expression was observed only in a limited subset of cancers, such as kidney chromophobe (KICH). Overall, these data indicate that CAD upregulation is a common feature across multiple human cancers.

**Figure 2:**
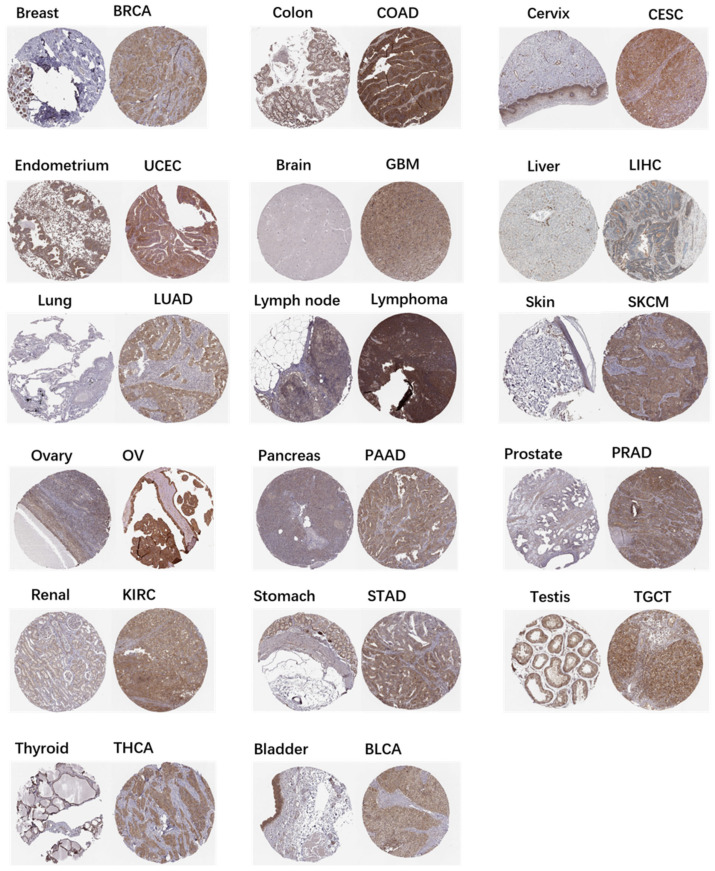
Protein expression levels of CAD across human pan-cancer. Representative immunohistochemistry (IHC) staining images derived from The Human Protein Atlas (HPA) demonstrate CAD protein expression in various normal human tissues and their corresponding cancer tissues. For each tissue type, the (**left** panel) displays normal tissue, while the (**right** panel) displays the corresponding cancer tissue. Brown staining indicates the presence of CAD protein.

To examine whether this pattern was also reflected at the protein level, we compared CAD immunohistochemical staining in tumor and corresponding normal tissues using data from the Human Protein Atlas (Figure 2). In general, CAD staining was weak to moderate in most normal tissues, whereas stronger staining intensity and a broader distribution of positive cells were observed in several tumor types, including lung, breast, liver, kidney, colorectal, gastric, and prostate cancers. These findings were broadly consistent with the transcriptomic results.

We further assessed the relationship between CAD expression and clinicopathological progression. Higher CAD expression was associated with more advanced clinical stage in KIRC, ACC, and KICH. In addition, increased CAD expression correlated with higher T stage and/or lymph node involvement in several tumor types, including liver hepatocellular carcinoma (LIHC), STAD, and BLCA (Figure S1).

Together, these findings suggest that CAD is broadly upregulated in human cancers and is associated with more aggressive clinicopathological characteristics.

### 3.2. CAD Exhibits Variable but Generally Favorable Diagnostic Performance Across Multiple Cancers

**Figure 3:**
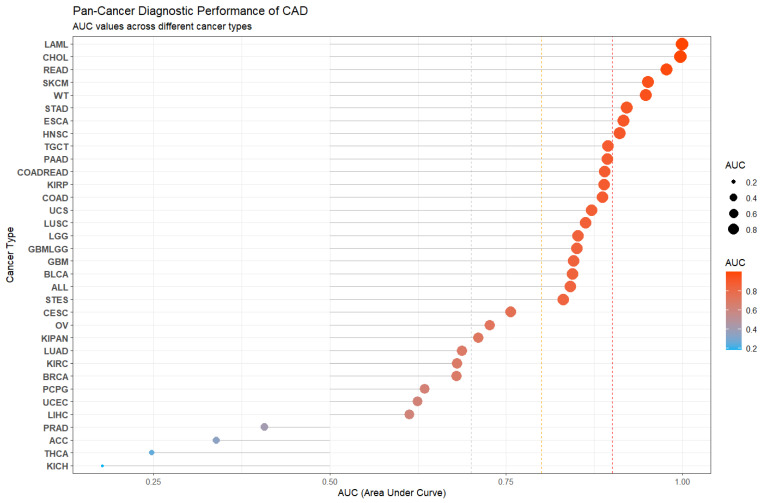
Pan-cancer diagnostic performance of the CAD model. The dot plot illustrates the Area Under the ROC Curve (AUC) values for the diagnostic classification of normal versus tumor tissues across various TCGA cancer types. The cohorts are ranked on the y-axis from lowest (bottom) to highest (top) diagnostic accuracy. Data distribution: AUC values indicate highly variable discriminatory power, ranging from poor diagnostic value in cohorts such as KICH and THCA (AUC < 0.3) to excellent diagnostic performance in malignancies like LAML and CHOL (AUC > 0.95). Visual elements: Both the size of the dots and the color gradient (ranging from blue for low values to red for high values) correspond to the magnitude of the AUC. The vertical dashed lines represent standard diagnostic reference thresholds (e.g., 0.7, 0.8, and 0.9) to easily categorize the predictive utility of CAD across different cancers.

We evaluated the ability of CAD expression to discriminate tumor tissues from normal tissues using receiver operating characteristic (ROC) analysis in the TCGA-GTEx pan-cancer cohort. As shown in Figure 3, the diagnostic performance of CAD varied across cancer types. The highest AUC values were observed in LAML and CHOL, approaching 1.0, while strong discriminatory ability (AUC > 0.9) was also found in STAD, SKCM, and READ. In addition, CAD showed moderate diagnostic performance (AUC 0.7–0.9) in several tumor types, including HNSC, ESCA, PAAD, and COAD. By contrast, limited diagnostic value was observed in certain cancers, such as KICH. These differences may reflect tumor-type-specific metabolic dependence on pyrimidine biosynthesis and differences in baseline CAD expression across corresponding normal tissues. Overall, these results indicate that CAD may have diagnostic relevance in selected cancer types, although its performance appears to be context dependent.

### 3.3. Genomic Alterations of CAD Across Cancers and Their Association with CAD Expression

Apart from gene expression, genetic variations and mutations such as activation of oncogenes or inactivation of tumor suppressor genes can also contribute to the occurrence and development of cancer [41]. We next characterized the genomic alteration profile of CAD across cancers using the cBioPortal and TIMER2.0 platforms. Analysis of TCGA data showed that CAD exhibited variable alteration frequencies across tumor types. Among them, uterine corpus endometrial carcinoma (UCEC) showed the highest overall alteration frequency (>12%), followed by skin cutaneous melanoma (SKCM) and stomach adenocarcinoma (STAD) (Figure S2B). Examination of alteration patterns further indicated that mutations were the predominant alteration type in UCEC, SKCM, and STAD, whereas copy number amplification was more common in ovarian serous cystadenocarcinoma (OV), esophageal carcinoma (ESCA), and LIHC (Figure S2A).

We further assessed the distribution of CAD mutation sites and found that somatic mutations were scattered throughout the coding sequence, without an obvious mutational hotspot (Figure S2C). These variants involved multiple functional regions of the CAD protein, including the CPSase, GATase, and DHOase domains, with missense mutations representing the predominant variant type (Figure S2D). This dispersed mutational pattern suggests that CAD alterations may be heterogeneous across tumor contexts rather than concentrated at a single recurrent site.

To explore the relationship between genomic alteration and transcriptional output, we next examined the association between CAD mRNA expression and copy number variation status (Figure S2E). In multiple cancer types, including LUAD, COAD/READ, and BRCA, samples with copy number amplification tended to show higher CAD expression than copy number-neutral or deletion samples. Similar patterns were observed in 17 of 33 cancer types, indicating that copy number gain may contribute to CAD overexpression in a substantial subset of tumors.

Finally, using the CAMOIP platform, we investigated whether CAD expression was associated with mutation patterns of established cancer-related genes. Patients were divided into high- and low-CAD expression groups using the median expression level as the cutoff. In BLCA, the high-CAD expression group showed significantly higher mutation frequencies of TP53 and RB1 (both *p* < 0.0001) (Figure S2F, left). In UCEC, elevated CAD expression was associated with mutations in PTEN and TP53 (both *p* < 0.0001) (Figure S2F, center), while in COAD, CAD expression was significantly associated with APC mutation status (*p* < 0.01) (Figure S2F, right). Together, these findings suggest that elevated CAD expression is associated with distinct genomic alteration patterns across cancers and may co-occur with mutations in key tumor suppressor pathways in specific tumor types.

### 3.4. High CAD Expression Is Associated with Poor Prognosis in Multiple Cancers

**Figure 4:**
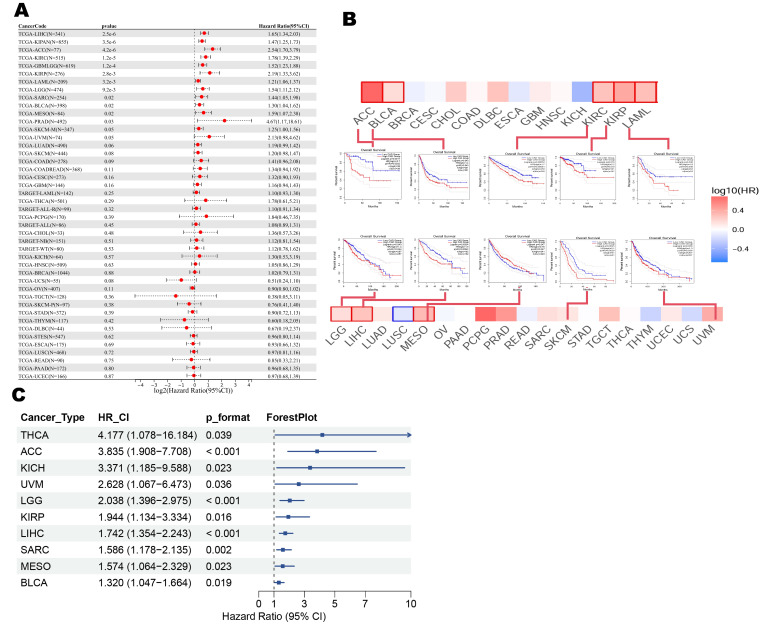
Survival analysis of CAD expression across cancers. (**A**) Comprehensive forest plot detailing the univariate Cox proportional hazards analysis of CAD expression for overall survival (OS) across TCGA and TARGET pan-cancer cohorts. The x-axis represents the log2-transformed Hazard Ratio [log2(HR)]. The cohorts are ranked by the statistical significance of the association (*p*-value). (**B**) Composite survival analysis across specific cancer types. The upper and lower heatmaps display the log10-transformed Hazard Ratios [Log10(HR)] for CAD expression across diverse malignancies, with red denoting increased risk and blue denoting protective trends. The linked Kaplan–Meier survival curves illustrate the significant differences in overall survival between CAD-High (red lines) and CAD-Low (blue lines) patient strata for representative cancer types (e.g., ACC, BLCA, LIHC). *p*-values were calculated using the Log-rank test. (**C**) Multivariable Cox proportional hazards regression analysis of CAD expression for overall survival (OS) across selected TCGA cancer cohorts. The model was adjusted for key clinical covariates, including age, gender, race, pathological stage, and tumor purity. Visual elements: Blue squares represent hazard ratio (HR) point estimates, and horizontal blue lines indicate the 95% confidence intervals (CI). The vertical dashed line at HR = 1 represents the null hypothesis (baseline of no effect); an HR > 1 (to the right of the line) indicates that elevated CAD expression is an independent risk factor for poor prognosis. Abbreviations: HR, hazard ratio; CI, confidence interval. The full clinical names for the displayed cancer type abbreviations (THCA, ACC, KICH, UVM, LGG, KIRP, LIHC, SARC, MESO, BLCA) are provided in Supplementary Table S1.

Given the widespread upregulation of CAD in malignant tissues, we next evaluated its association with patient survival across cancers. Survival analyses showed that high CAD expression was associated with worse overall survival (OS) in multiple tumor types, including ACC, LIHC, and KIRC (Figure 4A,B). In addition, elevated CAD expression was associated with shorter disease-free survival (DFS) in several cancers, such as KIRP and LGG (Figure S3).

To further assess whether CAD was independently associated with prognosis, multivariable Cox proportional hazards regression models were constructed using the TIMER2.0 survival module. CAD expression was analyzed together with all available clinical covariates, including age, gender, race, pathological stage, and tumor purity. For each model, complete-case analysis was applied, and cancer types where these covariates were adequately represented were included in the analysis. As shown in Figure 4C, CAD remained significantly associated with OS in several cancer types, including LIHC, KIRC, and ACC, after adjustment for these factors. Together, these findings suggest that elevated CAD expression is associated with unfavorable clinical outcomes across multiple cancers and may have prognostic relevance beyond established clinical variables.

### 3.5. CAD Expression Positively Correlates with Tumor Dedifferentiation Markers and Epitranscriptomic Regulators

We next assessed whether CAD expression was associated with tumor dedifferentiation by analyzing multiple stemness indices. Pearson correlation analysis showed that CAD expression was positively correlated with several stemness metrics, including DMPss, DNAss, ENHss, EREG.EXPss, EREG.METHss, and RNAss, across most TCGA cancer types (Figure S4A–F). Particularly consistent positive associations across all evaluated stemness indices were observed in malignancies such as KIPAN and HNSC. Overall, these findings suggest that elevated CAD expression is broadly associated with dedifferentiation-related features across cancers.

We further examined the relationship between CAD expression and RNA modification-related regulators. Using the Sangerbox platform, we analyzed the co-expression patterns between CAD and regulators of m6A, m1A, and m5C modification across pan-cancer cohorts (Figure S4G). Positive correlations predominated across many tumor types, and a substantial proportion of these associations were statistically significant, indicating that higher CAD expression was frequently accompanied by higher expression of RNA modification regulators. These results suggest that CAD expression is associated with RNA modification-related transcriptional patterns in multiple cancers, although the biological basis of this relationship requires further investigation.

### 3.6. Identification of a Core Co-Expression Network Associated with CAD-Related Cell Cycle and Metabolic Features

**Figure 5:**
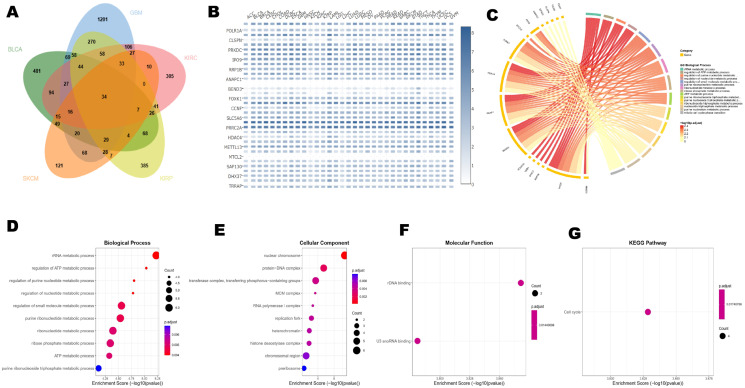
Enrichment analysis of genes co-expressed with CAD across multiple cancer types. (**A**) Venn diagram illustrating the overlap of CAD-co-expressed genes across five distinct tumor types: BLCA (green), SKCM (orange), KIRP (yellow), KIRC (red), and GBM (blue). The numerical values indicate the count of common and unique co-expressed genes. (**B**) Heatmap depicting the expression profiles of the 34 core intersecting CAD-co-expressed genes across various cancers. The color gradient (light to dark blue) reflects increasing expression levels. (**C**) Chord diagram visualizing the dynamic relationships between the CAD-co-expressed gene set and their significantly enriched Gene Ontology (GO) Biological Process terms (e.g., rRNA metabolic process, regulation of ATP metabolic process, and mitotic cell cycle phase transition). Ribbon widths correspond to the number of genes associated with each specific process. (**D**–**G**) Bubble plots detailing the GO and KEGG pathway enrichment analyses. The panels display significantly enriched (**D**) GO Biological Processes, © GO Cellular Components, (**F**) GO Molecular Functions, and (**G**) KEGG pathways (e.g., cell cycle). For all bubble plots, the *x*-axis represents the enrichment score [−log_10_(*p*-value)], node size indicates the gene count, and color gradient represents the adjusted *p*-value (p.adjust).

To further characterize the biological programs associated with CAD across cancers, we selected five representative tumor types—BLCA, GBM, KIRC, KIRP, and SKCM—based on their integrated associations with high CAD expression, stemness-related features, RNA modification-related patterns, and poor prognosis. For each cancer type, CAD co-expressed genes were identified and intersected, yielding a set of 34 shared co-expressed genes (Figure 5A).

We first examined the expression patterns of these 34 genes across 33 cancer types using a heatmap (Figure 5B). Many of these genes, including POLR1A, CLSPN, PRKDC, TRRAP, METTL13, and HDAC4, showed broadly elevated expression across multiple tumors.

To further explore the biological functions associated with this co-expression network, we performed Gene Ontology (GO) and Kyoto Encyclopedia of Genes and Genomes (KEGG) enrichment analyses. GO biological process analysis indicated that these genes were mainly enriched in processes related to nucleotide metabolism, ATP metabolic regulation, and rRNA metabolism (Figure 5D). In addition, GO cellular component and molecular function analyses showed enrichment in nuclear chromosome-related structures and the RNA polymerase I complex (Figure 5E,F). KEGG analysis identified significant enrichment in the cell cycle pathway (Figure 5G). The chord plot further illustrated the functional relationships among representative genes, including MTOR, PFAS, and PRKDC, across these enriched biological processes (Figure 5C).

Overall, these results suggest that CAD-associated co-expression networks are closely linked to cell-cycle regulation and anabolic metabolic processes across cancers. These patterns are broadly consistent with the established role of CAD in de novo pyrimidine biosynthesis, while also indicating that elevated CAD expression may occur within a broader transcriptional program related to tumor proliferation and metabolic demand [42,43].

### 3.7. Integrative Analysis Reveals Associations Between CAD, Pyrimidine Metabolism, and Cellular Dependency

To further characterize the metabolic features associated with CAD, we integrated transcriptomic, metabolomic, and genome-wide CRISPR screening data from 224 cancer cell lines obtained from the DepMap database.

**Figure 6:**
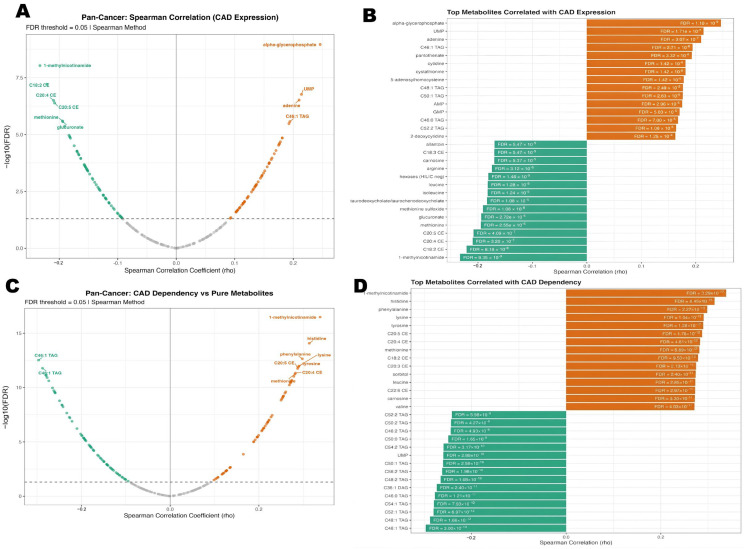
Integrated analysis of CAD-associated metabolic landscape and CRISPR gene dependency. (**A**) Volcano plot illustrating the Spearman correlation between CAD mRNA expression and intracellular metabolite abundance across cancer cell lines. Orange dots and green dots represent metabolites significantly positively and negatively correlated with CAD expression, respectively. (**B**) Bar chart detailing the top metabolites most strongly correlated with CAD expression. Alpha-glycerophosphate and select nucleotides (e.g., UMP, adenine, AMP, and GMP) exhibit the most significant positive correlations (orange bars). (**C**) Volcano plot displaying the Spearman correlation between baseline intracellular metabolite abundance and CAD CRISPR gene effect scores (Chronos). (**D**) Bar chart identifying the top metabolic predictors of CAD dependency. The green section (negative correlation) indicates that higher abundance of specific metabolites—predominantly triglycerides (TAGs) and UMP—correlates with more negative CRISPR scores (stronger dependency). Conversely, the orange section (positive correlation) reveals that higher levels of 1-methylnicotinamide and various amino acids (e.g., histidine, phenylalanine, lysine, and tyrosine) are associated with lower dependency. Note: In panel (**D**), a negative correlation implies that elevated baseline metabolite levels sensitize cells to CAD knockout. Statistical analysis: Correlation coefficients were calculated using Spearman’s rank correlation to account for potential non-normal distributions. Nominal *p*-values were adjusted for multiple testing using the Benjamini–Hochberg False Discovery Rate (FDR) method. FDR < 0.05 was considered statistically significant.

First, we assessed the relationship between CAD mRNA expression and metabolite abundance using Spearman correlation analysis (Figure 6A,B). CAD expression showed positive associations with several nucleotides and nucleotide-related metabolites, including uridine monophosphate (UMP), cytidine, adenine, adenosine monophosphate (AMP), and guanosine monophosphate (GMP), whereas the strongest positive correlation was observed for alpha-glycerophosphate. In contrast, several essential amino acids, including methionine, leucine, and isoleucine, as well as hexoses, were negatively correlated with CAD expression. In addition, selected lipid species, such as cholesterol esters (C18:2 CE and C20:4 CE), also showed inverse associations. Overall, these patterns suggest that elevated CAD expression is associated with a metabolic state characterized by increased nucleotide-related metabolites and altered nutrient utilization, consistent with the established biological function of CAD as a nucleotide metabolism enzyme [44].

We next analyzed the functional dependency of cancer cell lines on CAD across lineages (Figure S5). CAD dependency scores were below zero in approximately 30 cancer lineages, with median values largely ranging from −0.5 to −1.0, indicating that CAD is broadly required for the fitness of many tumor cell lines. By comparison, some non-malignant lineages, such as fibroblast and adrenal cell models, exhibited relatively weaker CAD dependency.

To further explore which metabolic features define the selective vulnerability of tumor cells to CAD loss, we analyzed the correlation between metabolite abundance and CAD Gene Effect Scores (Figure 6C,D). Because lower Chronos scores indicate stronger dependency, negative correlations suggest that higher metabolite abundance is associated with greater reliance on CAD [45]. Under this framework, higher basal levels of UMP, together with enrichment of several triglycerides (TAGs) and diacylglycerols (DAGs), including C46:1 TAG and C50:1 TAG, were associated with increased CAD dependency. In contrast, higher levels of certain amino acids, such as histidine, phenylalanine, and lysine, as well as cholesterol esters including C20:4 CE, were associated with lower CAD dependency. These findings suggest that CAD dependency is more pronounced in cell lines with metabolic features consistent with elevated nucleotide demand and active biosynthetic metabolism [46].

Taken together, these integrative analyses indicate that CAD expression and CAD dependency converge on metabolic states related to nucleotide metabolism and cellular biosynthetic demand. These observations are consistent with the established role of CAD in de novo pyrimidine biosynthesis and further suggest that CAD may be particularly important in tumor cells operating under heightened anabolic requirements, although the mechanistic basis of these associations will require direct experimental investigation [47].

### 3.8. CAD Expression Is Associated with Immunosuppressive Features and Altered Immune Infiltration Patterns

**Figure 7:**
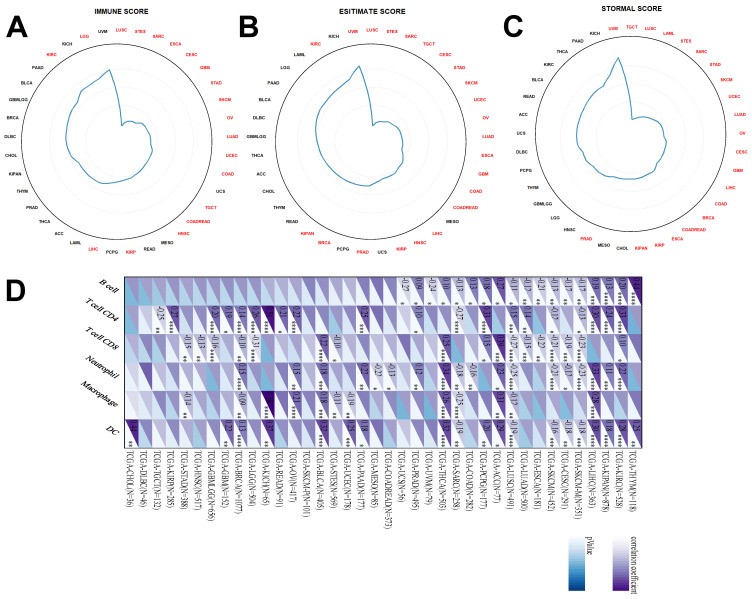
Correlation analysis between CAD and immune infiltration in multiple cancers. (**A**–**C**) Radar plots illustrating the correlation between CAD expression and tumor microenvironment metrics, including the (**A**) ImmuneScore, (**B**) ESTIMATEScore, and (**C**) StromalScore across various TCGA cohorts. The cancer types are labeled around the perimeter. Cancer types marked in red indicate a statistically significant correlation (*p* < 0.05). The blue lines depict the overall correlation trend across the evaluated malignancies. (**D**) Heatmap detailing the correlation between CAD expression and the infiltration levels of specific immune cell types (B cells, CD4+ T cells, CD8+ T cells, neutrophils, macrophages, and dendritic cells). Color coding: Positive correlations are mapped in purple, while negative correlations are depicted in blue. The intensity of the color reflects the magnitude of the correlation coefficient. Statistical significance is indicated by asterisks (* *p* < 0.05, ** *p* < 0.01, *** *p* < 0.001, **** *p* < 0.0001).

To investigate the relationship between CAD expression and the tumor microenvironment (TME), we applied the ESTIMATE algorithm to evaluate ImmuneScore, StromalScore, and ESTIMATEScore across pan-cancer cohorts, and summarized the correlations in radar plots (Figure 7A–C).

Across multiple tumor types, CAD expression was significantly negatively correlated with ESTIMATE, Immune, and Stromal scores, including in LUSC, STAD, and SKCM, suggesting that CAD-high tumors tend to exhibit reduced non-tumor microenvironmental components in these cancer types. In contrast, in selected tumors such as KICH, KIRC, and PAAD, CAD expression showed positive or near-positive correlations with StromalScore, indicating that the relationship between CAD and stromal components may vary across tumor contexts.

We next examined the association between CAD expression and immune cell infiltration (Figure 7D). CAD expression was positively correlated with immune cell abundance in some tumor types, including LUAD, KIRC, and LIHC (*p* < 0.001), whereas negative correlations were observed in LUSC, SKCM, and STAD [48,49]. Notably, among tumor types showing inverse correlations, CD8+ T-cell infiltration appeared to be particularly reduced. Overall, these findings indicate that the relationship between CAD expression and immune infiltration is heterogeneous across cancers, but in several tumor types, elevated CAD expression is associated with a relatively immune-suppressed microenvironment [48,49].

Taken together, these analyses suggest that CAD expression is linked to distinct tumor microenvironmental features across cancers. In particular, the inverse associations observed with immune-related scores and CD8+ T-cell infiltration in several tumor types raise the possibility that CAD-high tumors may be associated with less favorable immune contexts. However, these observations are based on computational inference and correlation analysis, and the underlying biological mechanisms remain to be determined.

### 3.9. The Multi-Cohort Validated Predictive Potential of CAD in Immunotherapy Response

**Figure 8:**
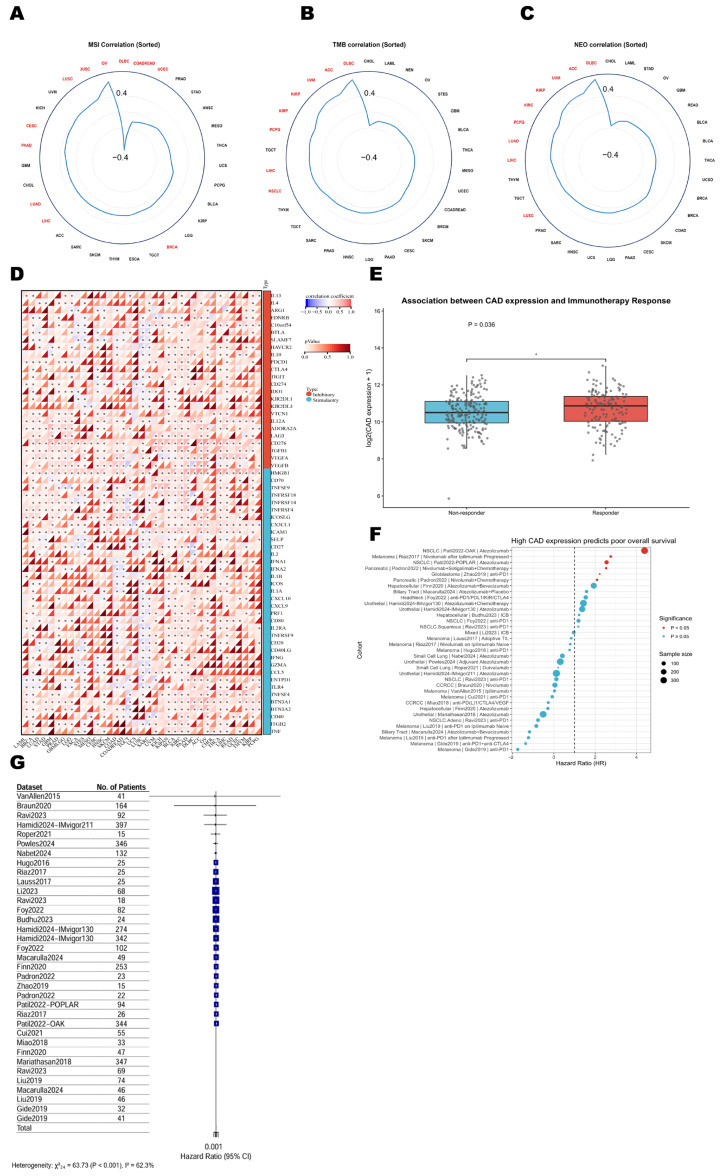
Correlation analysis between CAD and immune checkpoint blockade proteins. (**A**–**C**) Radar plots illustrating the correlation between CAD expression and key immunogenicity metrics: (**A**) microsatellite instability (MSI), (**B**) tumor mutational burden (TMB), and (**C**) neoantigen burden (NEO) across various cancers. Cancer types are labeled around the perimeter, with statistically significant correlations (*p* < 0.05) highlighted in red. The blue lines indicate the correlation coefficients, while the inner numerical values (0.4 and −0.4) represent the radial axis scale. (**D**) Comprehensive heatmap detailing the correlation between CAD expression and an array of immune checkpoint blockade (ICB)-related genes across multiple cancer types. Color coding: The gradient represents the correlation coefficient, where red indicates positive correlations and blue indicates negative correlations. Statistical significance is exclusively denoted by asterisks (*). (**E**) Box plot comparing CAD expression levels (log2-transformed) between patients classified as Responders (red box) and Non-responders (blue box) to ICB therapy. Elevated CAD expression is significantly associated with a positive initial immunotherapy response (*p* = 0.036). (**F**) Bubble plot summarizing the prognostic value of high CAD expression across multiple immunotherapy cohorts. To accommodate the display scale, the x-axis represents the log-transformed Hazard Ratio (log HR). Bubble size corresponds to the sample size of each cohort, while color denotes statistical significance (red indicates *p* < 0.05; blue indicates *p* ≥ 0.05). The vertical dashed line represents HR = 1. (**G**) Detailed forest plot and meta-analysis of CAD expression concerning immunotherapy survival outcomes. The plot displays the specific point estimates (squares) and horizontal lines representing the 95% confidence intervals (CI) for each dataset. The bottom section reports the heterogeneity statistics (*χ*^2^, *I*^2^) and the pooled overall significance. Statistical analysis: Survival outcomes (Overall Survival, Progression-Free Survival) were assessed using Kaplan–Meier curves with the log-rank test. For the meta-analysis, pooled Hazard Ratios (HR) and 95% Confidence Intervals (CI) were calculated using a random-effects model to account for inter-study heterogeneity (*I*^2^). The predictive performance for immunotherapy was evaluated using Area Under the ROC Curve (AUC). * *p* < 0.05.

To further evaluate the potential clinical relevance of CAD in immunotherapy, we first analyzed the associations between CAD expression and three commonly used immunotherapy-related biomarkers, including tumor mutational burden (TMB), microsatellite instability (MSI), and neoantigen burden (NEO) (Figure 8A–C) [50]. Radar plot analyses showed that CAD expression was significantly correlated with these immunogenicity-related indicators in multiple cancer types. Notably, CAD showed concurrent positive correlations with TMB, MSI, and NEO burden in cancers such as DLBC, KIRC, and ACC, whereas negative associations were observed in others, including LIHC and LUAD. These findings suggest that the relationship between CAD expression and immunogenicity-related features is tumor-type dependent, and that CAD-high tumors may coincide with a more immunogenic genomic background in selected cancer contexts.

Subsequently, we created a co-expression heatmap of CAD and immune checkpoint genes. The expression level of CAD was correlated with two broad categories of immune-related genes: immune checkpoints (such as PD-L1/CD274, CTLA4, TIGIT) and immune stimulatory factors (such as CD28, ICOS, IL2, TNF). The results showed that in multiple cancer types (Figure 8D), such as UVM, KICH, LIHC, PAAD, etc., there was a very strong positive correlation with CD274 (PD-L1) (deep red). Similarly, CTLA4 also showed widespread high expression consistency. At the same time, target genes such as TIGIT, LAG3, and IDO1 were also upregulated synchronously with CAD. We also found that in UVM, KICH, and KIRC, the expression of CAD showed a significant positive correlation with the entire immune regulatory network. This further confirmed the conclusion in the radar chart that these tumors belong to the “immune-inflammatory type”. In DLBC, there was a very strong correlation with inhibitory checkpoints (upper part), indicating that it may be used to predict the sensitivity to targeted therapy.

To further assess the clinical relevance of CAD in immunotherapy-treated patients, we analyzed public cohorts receiving immune checkpoint blockade (ICB). In the ROCplotter dataset, using pre-treatment biopsy samples, CAD expression was significantly higher in responders than in non-responders (*p* = 0.036, Figure 8E), suggesting a potential association between elevated CAD expression and initial ICB response. However, analysis of the CIDE database showed that high CAD expression was associated with reduced overall survival in several ICB-treated cohorts (Figure 8F). Similar trends were observed in individual cohorts such as Riaz2017, POPLAR, and OAK, in which elevated CAD expression was associated with poorer survival outcomes [51,52]. To further quantify this association, we screened 53 independent immunotherapy cohorts and included 25 cohorts with complete overall survival data in a meta-analysis (Figure 8G). High CAD expression was associated with an increased risk of death, with a pooled hazard ratio (HR) of 1.42 (95% CI: 1.19–1.70, *p* < 0.001).

Taken together, these findings suggest that CAD expression may be associated with both immunogenicity-related features and clinical outcomes in immunotherapy-treated patients. The observation that higher pre-treatment CAD expression was linked to response in one dataset, yet to poorer overall survival across multiple cohorts, points to a potentially paradoxical role of CAD in immunotherapy. One possible interpretation is that CAD-high tumors may combine greater potential for initial immune recognition with a less favorable biological context for durable antitumor control. However, these findings are based on retrospective and correlative analyses, and the mechanisms underlying these associations require further investigation.

## 4. Discussion

In this study, we conducted the first systematic analysis of CAD across different cancer types. We used multi-omics data to reveal its central role in tumorigenesis, metabolic changes, and immune regulation. Our findings suggest that CAD has potential as a valuable diagnostic and prognostic candidate marker. More importantly, we also achieved this by combining metabolomics data, immunotherapy response data with large-scale clinical cohorts. Its role in immunotherapy was also explored.

Early detection and accurate risk stratification remain paramount challenges in clinical oncology. The alteration of gene expression is one of the key signals for detecting cancer [53]. In this study, CAD, as a nucleotide metabolism enzyme, demonstrates potential as a clinical diagnostic biomarker. The ubiquitous upregulation of CAD at both transcriptional and translational levels across diverse malignancies suggests its potential association with tumor initiation and progression. Crucially, these expression changes were found to be closely linked to underlying genomic alterations. High CAD mRNA expression was significantly correlated with copy number variations, and its expression level was also positively associated with the mutation status of core tumor suppressor genes, such as TP53 and PTEN. Furthermore, CAD exhibited notable diagnostic performance in our subsequent ROC analyses. Additionally, a positive correlation between CAD expression and advanced clinicopathological stages was observed in the pan-cancer analysis, further suggesting its potential utility in clinical diagnosis and continuous disease monitoring.

Our study also suggests that CAD shows promise as a candidate prognostic marker. Survival analysis showed that in cancers such as LIHC, KIRC, and ACC, patients with high expression of CAD had significantly shorter overall survival periods.

We also examined the stemness characteristics. We found that CAD was positively correlated with various stemness indices (such as DNAss and RNAss) within the pan-cancer range (Figure S4A–F). This indicates that CAD may support the maintenance of stem cell-like characteristics in cancer cells, which could potentially contribute to tumor invasiveness and make it more difficult to treat [54,55]. Additionally, the expression level of CAD was positively correlated with RNA modification regulatory factors (such as m6A and m5C). The co-expression of CAD with m6A/m5C regulatory factors suggests that CAD expression might be subject to epigenetic regulation, which could potentially enhance the stability or translation efficiency of its mRNA [56,57], thereby further amplifying its carcinogenic effect at the post-transcriptional level.

The co-expressed gene analysis of CAD showed that the functional pathways of its co-expressed genes were mainly focused on cell cycle and energy metabolism. This enrichment suggests that CAD may be involved in tumorigenesis, tumor progression, and metabolic reprogramming. We also observed the relationship between CAD and immune cells within the tumor. There was some heterogeneity in the results. In THYM and LIHC, High CAD levels are associated with higher levels of immune cell infiltration, whereas the opposite pattern is observed in LUSC and SKCM (Figure 7D). This suggests that CAD influences the tumor environment differently across cancers [58]. In addition, CAD was strongly associated with TMB, MSI, and NEO. High levels of these markers generally imply that the tumor is more readily recognized by the immune system [47,59]. Importantly, CAD exhibited a positive correlation with key checkpoint genes, including PD-1 and PD-L1 (Figure 8D). This implies that CAD may be associated with an environment that allows tumors to evade the immune system [60].

Our analysis in the field of immunotherapy revealed a unique phenomenon: patients who responded to the treatment had a higher CAD level than those who did not respond (*p* = 0.036). This is consistent with the co-expression pattern of immune checkpoints, suggesting that the prognosis of immunotherapy is good. However, after integrating external immunotherapy cohorts, it was shown that the high expression of CAD is a risk factor for poor prognosis. To explain this phenomenon, we proposed several hypotheses based on our transcriptomic and metabolomic findings:

(1) CAD may drive rapid tumor growth and frequent mutations. Easy recognition by the immune system. However, the tumor is too aggressive and ultimately leads to a poor prognosis [61].
(2) Metabolic competition: Combined with our metabolomic analysis, CAD consumes large amounts of essential nutrients to synthesize DNA. T cells likewise require this material base to support their function. Cancer cells might deplete nutrients through rapid proliferation, potentially rendering immune cells unable to maintain normal function. This “metabolic competition” helps tumors achieve immune escape [62,63].
(3) Genomic complexity: Elevated CAD expression may be associated with increased chromosomal instability (CIN). Although CIN can drive immune recruitment via the cGAS-STING pathway or other mechanisms, chronic and high-level CIN often triggers secondary immunosuppressive pathways. In this context, a high TMB may temporarily mask the underlying immunosuppressive effects induced by pervasive CIN, thereby contributing to the phenomenon we observed [64];
(4) Adaptive immune resistance: The strong positive correlation between CAD and inhibitory checkpoints (e.g., PD-1, PD-L1) suggests the activation of adaptive immune resistance. While these checkpoints are targets for therapy, their upregulation also reflects the tumor’s intrinsic ability to suppress T-cell activity. Thus, while CAD-high tumors may be more “responsive” to checkpoint blockade due to the presence of these targets, the underlying tumor biology remains highly resistant, potentially offsetting long-term clinical benefits [65,66].

Given CAD’s role as a nucleotide metabolism enzyme, and in line with the metabolic competition hypothesis we proposed, we further suggest that the decoupling of initial immunotherapy sensitivity from long-term survival in CAD-high tumors may reveal metabolic interactions within the TME. As a trifunctional enzyme complex, CAD governs the rate-limiting steps of de novo pyrimidine synthesis, a process strictly dependent on the continuous influx of glutamine and aspartate. We postulate that the accelerated anabolism required for tumor proliferation in CAD-overexpressing malignant cells disproportionately depletes these specific amino acids, thereby causing a spatial uneven distribution of these amino acids within the TME. This spatial uneven distribution of amino acids affects the energy acquisition of tumor-infiltrating lymphocytes (TILs). Effector CD8^+^ T cells rely on exogenous glutamine not only for nucleotide synthesis but also to fuel the tricarboxylic acid (TCA) cycle via glutaminolysis, which is crucial for maintaining mTORC1 signaling and c-Myc-dependent metabolic reprogramming upon antigen stimulation. Consequently, the glutamine-deprived microenvironment created by CAD hyperactivation is consistent with impaired T cell clonal expansion and the production of effector cytokines. Furthermore, prolonged metabolic stress under nutrient-restricted conditions forces TILs to undergo metabolic and epigenetic adaptations, ultimately leading to terminal exhaustion.

This proposed hypothesis explains our co-expression findings, namely that CAD expression is synchronized with the upregulation of key coinhibitory receptors, including PD-1, CTLA-4, and TIGIT. Ultimately, although CAD-induced genomic instability (reflected by high TMB and NEO) may transiently enhance tumor immunogenicity and predict initial ICB recognition, the global metabolic competition blunts the sustained cytotoxic T cell response. This distinct metabolic–immune antagonism provides a theoretical basis for CAD as a potential contributor to adaptive immune evasion and poor clinical prognosis.

Based on our computational framework, CAD exhibits a degree of translational potential as a multi-dimensional clinical biomarker and therapeutic target. Critically, the exceptional diagnostic accuracy of CAD observed in specific malignancies (such as LAML and CHOL) suggests that standardized immunohistochemistry (IHC) quantification in baseline tumor biopsies warrants exploration for early detection and patient stratification. Therapeutically, combining small-molecule CAD inhibitors (such as PALA [66]) with immune checkpoint blockers (ICBs) represents a promising conceptual strategy to alleviate microenvironmental nutrient competition and counteract nucleotide-driven proliferation [67,68]. However, these preliminary insights are strictly theoretical and fundamentally require rigorous future wet-lab validation and prospective clinical trials before actual clinical implementation.

## 5. Research Limitations

Despite the comprehensive multi-omics approach, several limitations of this study must be acknowledged. First, the current analyses are fundamentally computational, correlative, and derived entirely from retrospective public datasets. Therefore, causal inference between CAD overexpression and immune evasion cannot be established based solely on these data. Second, inherent sampling and annotation biases, as well as platform heterogeneity across the integrated public datasets, cannot be entirely eliminated. Third, the immune associations and hypotheses proposed herein—particularly regarding CAD-associated metabolic competition and T-cell exhaustion—remain highly speculative. There is a critical need for subsequent mechanistic experiments, such as in vitro and in vivo functional assays (e.g., CRISPR-mediated CAD knockout models coupled with isotope-tracing metabolomics), to elucidate the precise metabolic–immune axis. Finally, before CAD can be considered a clinically actionable biomarker, these computational findings require rigorous validation in independent, large-scale, and prospective clinical cohorts.

## 6. Conclusions

This study systematically explores the multifaceted profile of CAD across diverse malignancies. Our computational analyses reveal that elevated CAD expression is closely linked to advanced clinical stages, enhanced stemness characteristics, and adverse prognostic outcomes. Furthermore, by observing the decoupling between initial immunotherapy sensitivity and long-term survival benefits, we hypothesize a potential mechanism of metabolic–immune antagonism within the tumor microenvironment. Ultimately, our findings suggest that CAD may represent a promising candidate biomarker associated with cancer progression and immunotherapy-related features, warranting further experimental and clinical validation.

## Data Availability

The original contributions presented in the study are included in the article/Supplementary Material. The datasets analyzed in this study were obtained from publicly available repositories, including: TCGA and GTEx: Accessed via the UCSC Xena browser (https://xenabrowser.net/) and GEPIA2 (http://gepia2.cancer-pku.cn/). Human Protein Atlas (HPA): Protein expression data and IHC images were retrieved from https://www.proteinatlas.org/. DepMap: CRISPR and metabolomics data were downloaded from the Cancer Dependency Map portal (https://depmap.org/portal/ accessed on 25 December 2025). cBioPortal: Genetic alteration data were sourced from https://www.cbioportal.org/. Immunotherapy Cohorts: External validation data were obtained through the CIDE website (https://cide.ccr.cancer.gov/ accessed on 25 December 2025) and ROC PLOTTER. Further inquiries can be directed to the corresponding authors.

## Supplementary Materials

The following supporting information can be downloaded at https://www.mdpi.com/article/10.3390/biomedicines14061218/s1, Figure S1: Association of CAD expression with clinicopathological stages across multiple cancers. Figure S2: Analysis of Distinct Genomic Characteristics of CAD Across Various Cancers. Figure S3: Forest plot of CAD expression associated with disease-free survival (DFS) in pan-cancer cohorts. Figure S4: Correlation analysis between CAD expression and cancer stemness or RNA modifications. Figure S5: CAD gene dependency across diverse cancer cell lineages. Table S1: Abbreviations and full names of the 33 cancer types in the TCGA pan-cancer cohort. Table S2: Multivariable Cox regression analysis results for CAD expression across TCGA cancer cohorts.
