# Supplementary material for: A Pan-Cancer Multi-Omics Analysis of CAD: Integrating CRISPR and Metabolomics Data to Unravel the Metabolic–Immune Axis and Immunotherapy Response
Source: Biomedicines. 2026 May 28;14(6):1218. doi: 10.3390/biomedicines14061218 (PMC13297017; doi:10.3390/biomedicines14061218)
Supplement: Supplementary file 1 [file biomedicines-14-01218-s001.zip › Supplementary Table S2.pdf]

| Cancer Type | Hazard Ratio (95% CI) | P-value | Status (for final analysis) |
|---|---|---|---|
| ACC | 3.835 (1.908–7.708) | < 0.001 | Included |
| BLCA | 1.320 (1.047–1.664) | 0.019 | Included |
| BRCA | 1.189 (0.925–1.528) | 0.177 | Included |
| CESC | 1.372 (0.942–1.997) | 0.099 | Included |
| CHOL | 1.060 (0.502–2.240) | 0.879 | Included |
| COAD | 1.068 (0.705–1.619) | 0.756 | Included |
| DLBC | 2.381 (0.407–13.915) | 0.336 | Excluded (Low events) |
| ESCA | 0.737 (0.481–1.130) | 0.162 | Included |
| GBM | 0.948 (0.797–1.126) | 0.542 | Included |
| HNSC | 0.983 (0.803–1.203) | 0.866 | Included |
| KICH | 3.371 (1.185–9.588) | 0.023 | Excluded (Sparse events) |
| KIRC | 1.326 (0.956–1.840) | 0.091 | Included |
| KIRP | 1.944 (1.134–3.334) | 0.016 | Included |
| LGG | 2.038 (1.396–2.975) | < 0.001 | Included |
| LIHC | 1.742 (1.354–2.243) | < 0.001 | Included |
| LUAD | 1.052 (0.870–1.273) | 0.598 | Included |

| | | | |
|---|---|---|---|
| LUSC | 0.961 (0.778–1.186) | 0.708 | Included |
| MESO | 1.574 (1.064–2.329) | 0.023 | Included |
| OV | 0.781 (0.594–1.028) | 0.078 | Included |
| PAAD | 0.953 (0.646–1.408) | 0.811 | Included |
| PCPG | 2.368 (0.492–11.396) | 0.282 | Excluded (Low events) |
| PRAD | 2.16e+20 (Inf) | 1 | Excluded (Unstable) |
| READ | 0.674 (0.253–1.798) | 0.431 | Included |
| SARC | 1.586 (1.178–2.135) | 0.002 | Included |
| SKCM | 1.063 (0.846–1.336) | 0.599 | Included |
| STAD | 0.782 (0.606–1.008) | 0.058 | Included |
| TGCT | 0.011 (0.000–Inf) | 1 | Excluded (Unstable) |
| THCA | 4.177 (1.078–16.184) | 0.039 | Included |
| THYM | 0.535 (0.162–1.772) | 0.306 | Excluded (Low events) |
| UCEC | 1.139 (0.792–1.639) | 0.481 | Included |
| UCS | 0.593 (0.293–1.200) | 0.146 | Included |
| UVM | 2.628 (1.067–6.473) | 0.036 | Included |

**Supplementary Table S2. Multivariable Cox regression analysis results for CAD expression across TCGA cancer cohorts.** Multivariable Cox regression models were performed for all 33 TCGA cancer types. Cohorts showing extreme HR values or model instability (e.g., PRAD, TGCT) due to sparse outcome events or limited sample sizes were

excluded from the final interpretation to ensure statistical reliability.
