# Supplementary figures and images for: A Pan-Cancer Multi-Omics Analysis of CAD: Integrating CRISPR and Metabolomics Data to Unravel the Metabolic–Immune Axis and Immunotherapy Response
Source: Biomedicines. 2026 May 28;14(6):1218. doi: 10.3390/biomedicines14061218 (PMC13297017; doi:10.3390/biomedicines14061218)

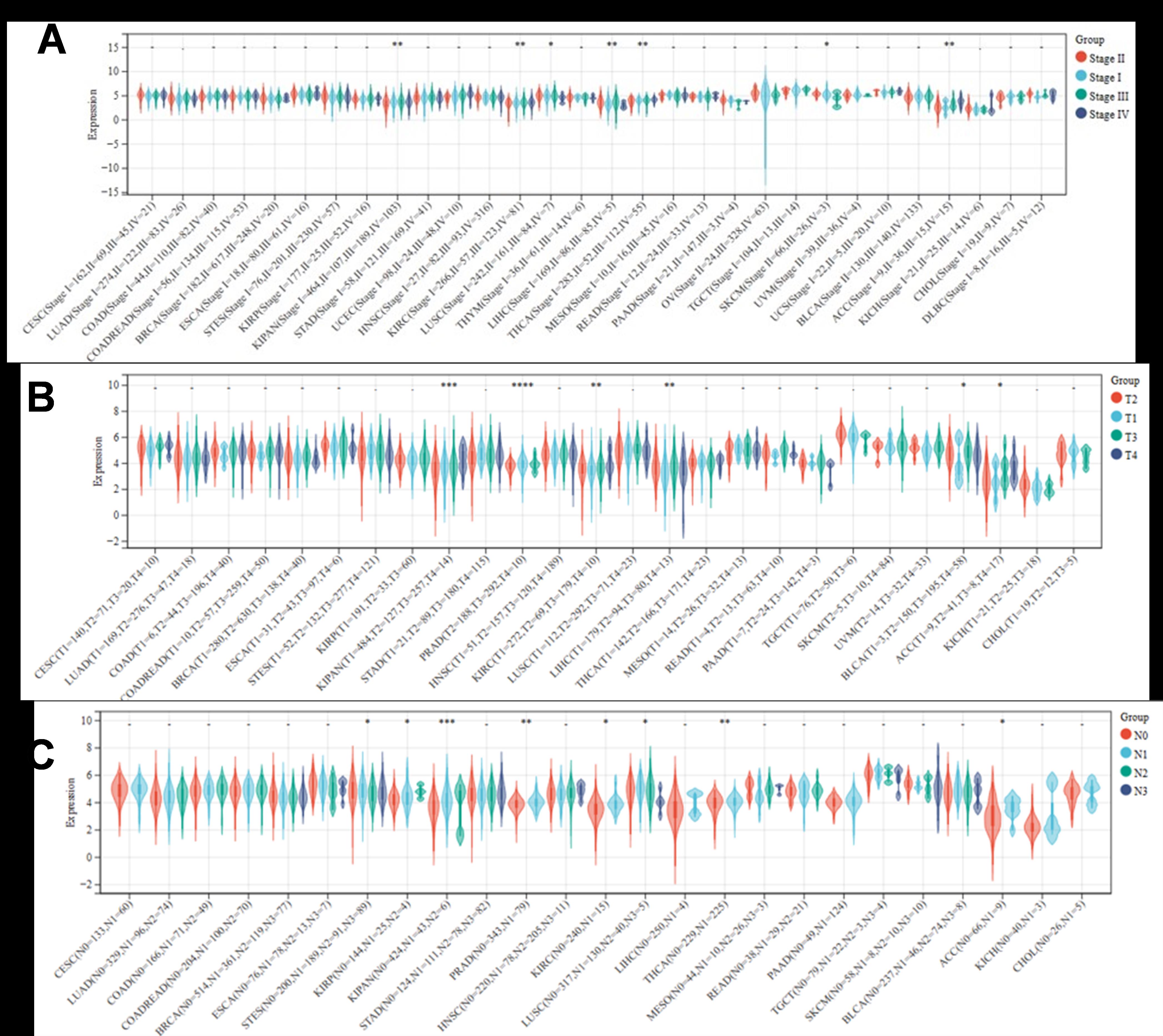

Supplement: Supplementary file 1 [file biomedicines-14-01218-s001.zip › figureS1/figureS1.jpg]

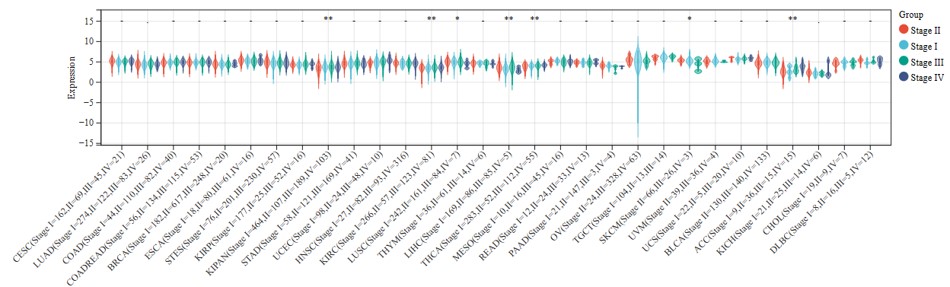

Supplement: Supplementary file 1 [file biomedicines-14-01218-s001.zip › figureS1/figureS1A.jpg]

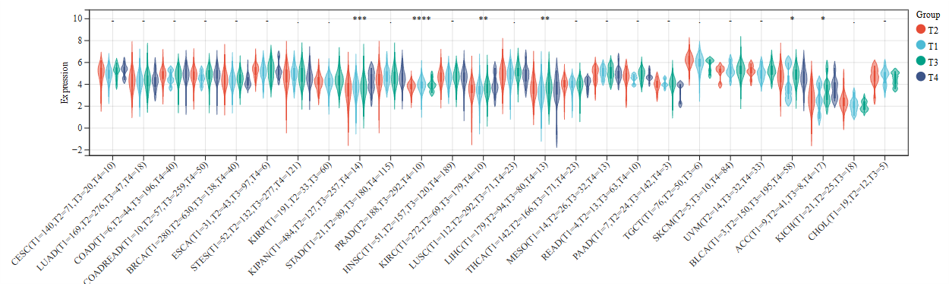

Supplement: Supplementary file 1 [file biomedicines-14-01218-s001.zip › figureS1/figureS1B.JPG]

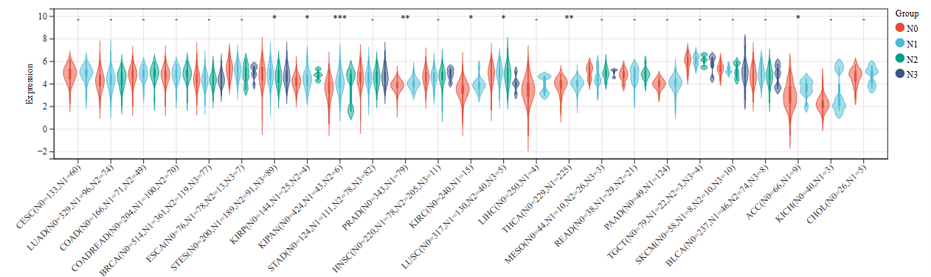

Supplement: Supplementary file 1 [file biomedicines-14-01218-s001.zip › figureS1/figureS1C.jpg]

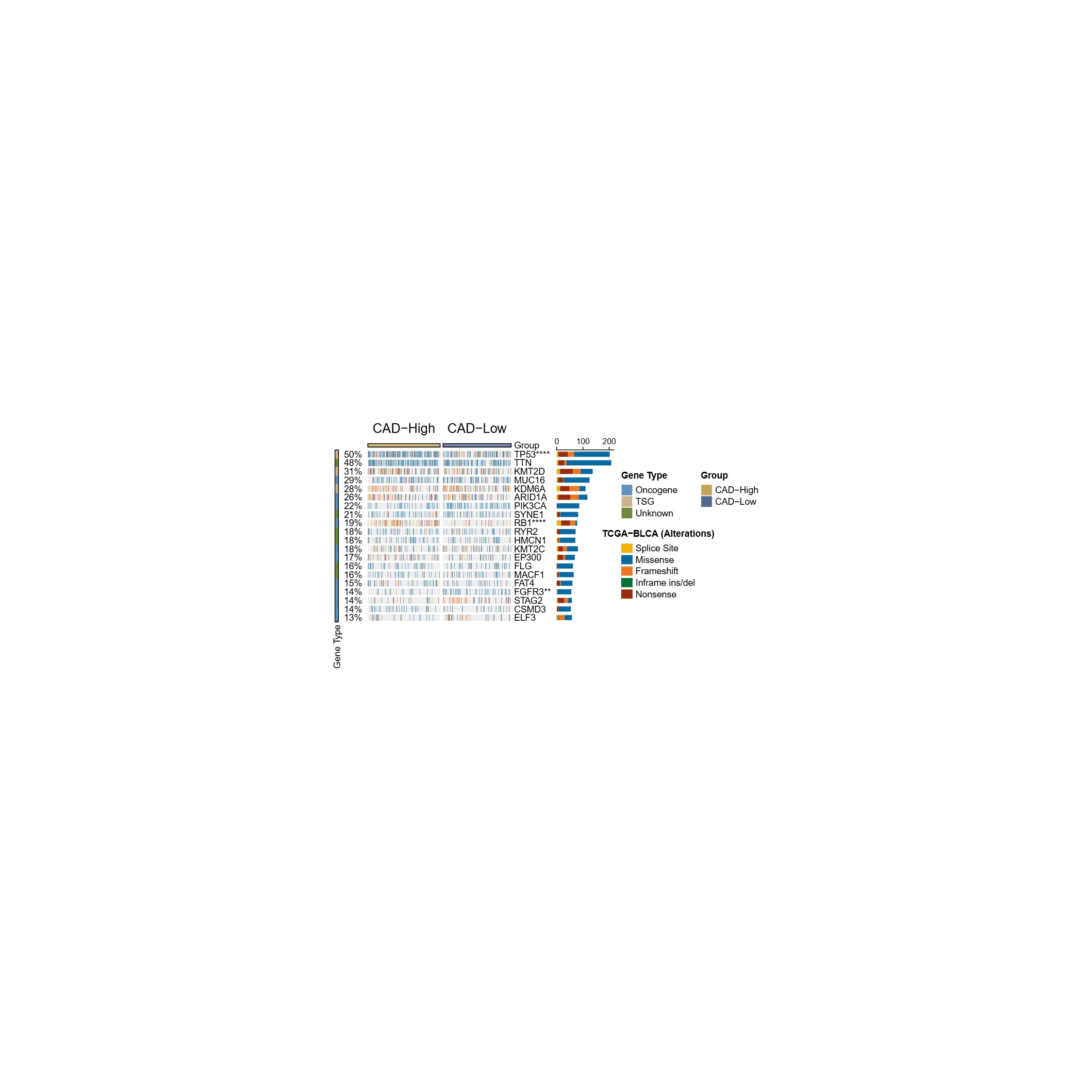

Supplement: Supplementary file 1 [file biomedicines-14-01218-s001.zip › figureS2/BLCA.jpg]

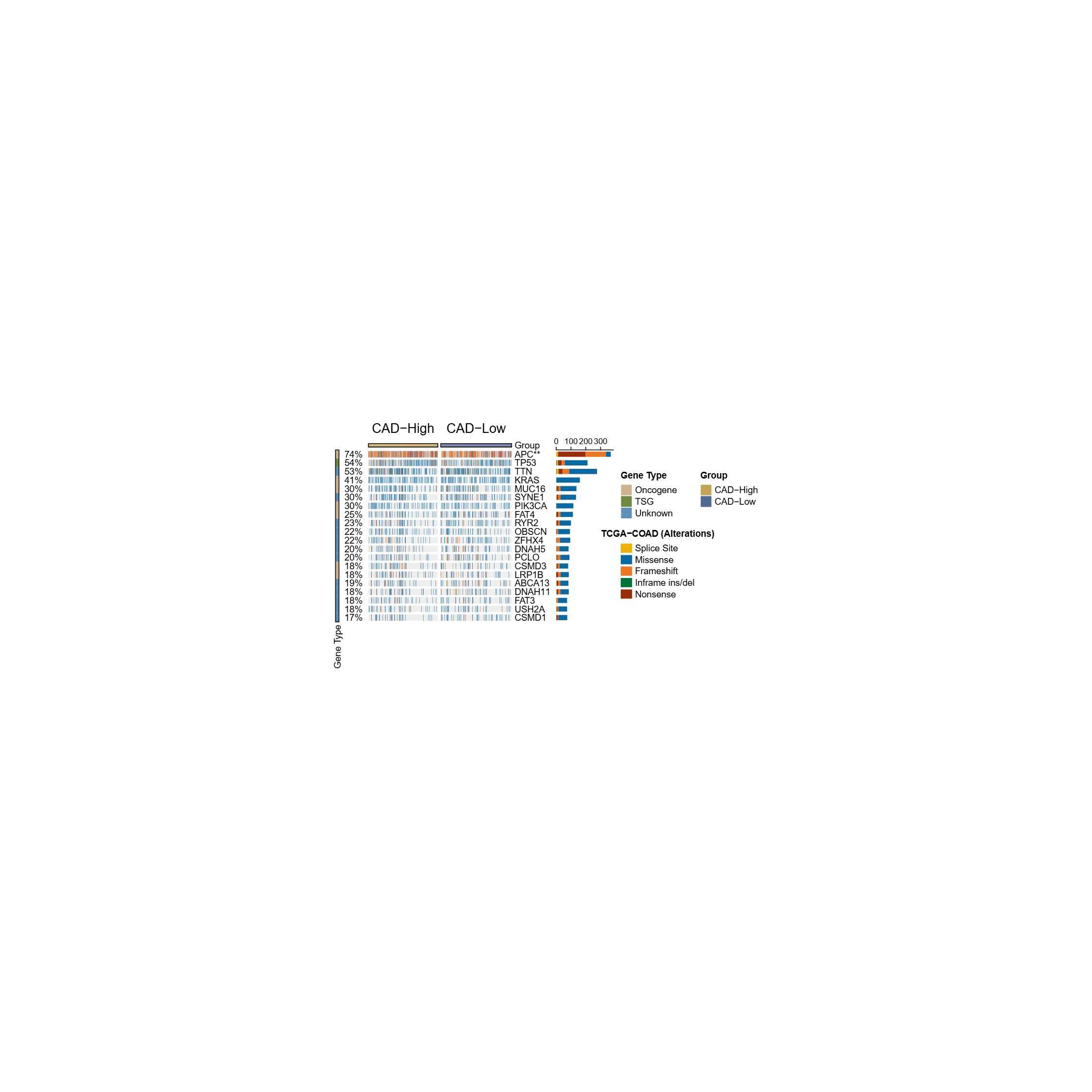

Supplement: Supplementary file 1 [file biomedicines-14-01218-s001.zip › figureS2/coad.jpg]

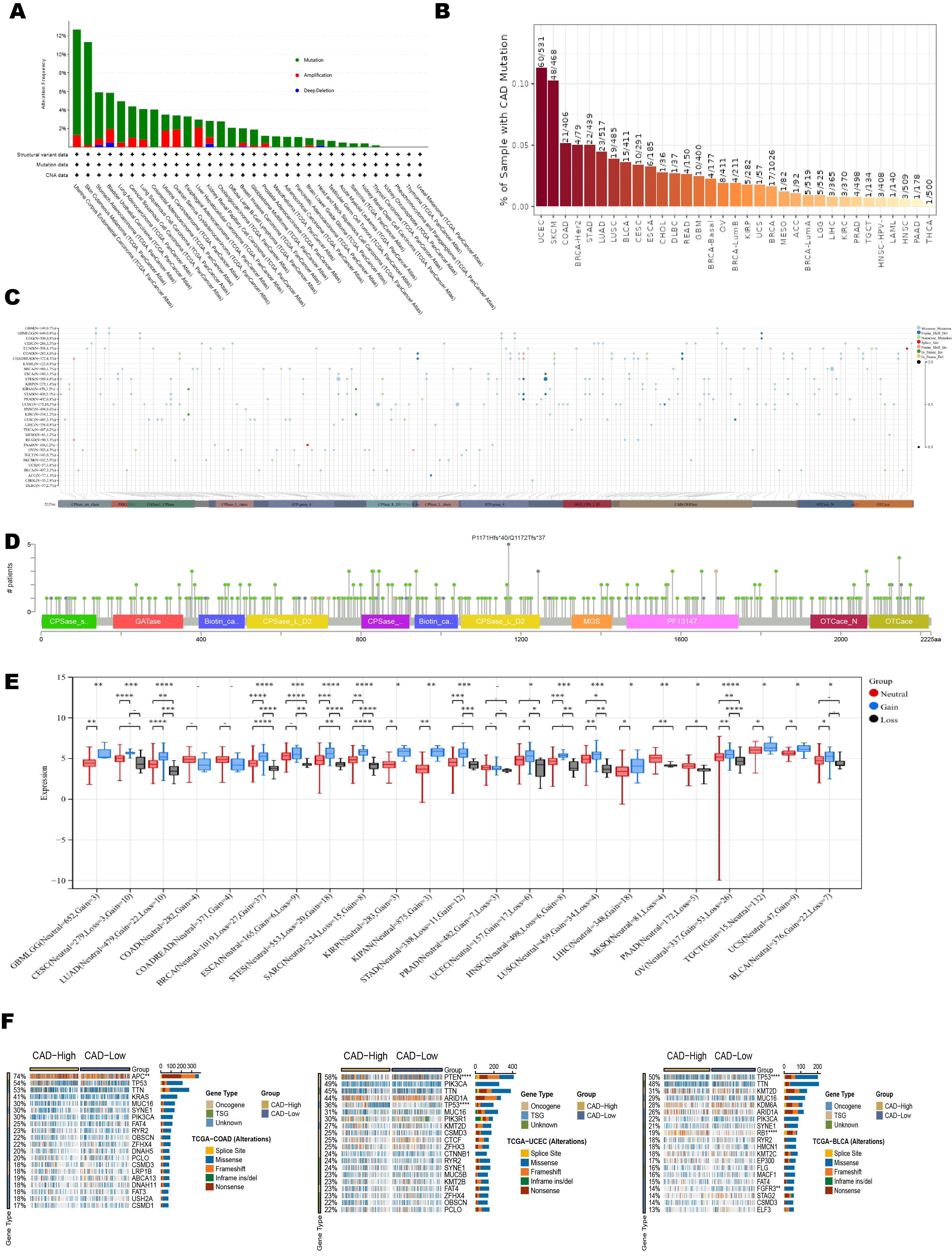

Supplement: Supplementary file 1 [file biomedicines-14-01218-s001.zip › figureS2/figureS6.jpg]

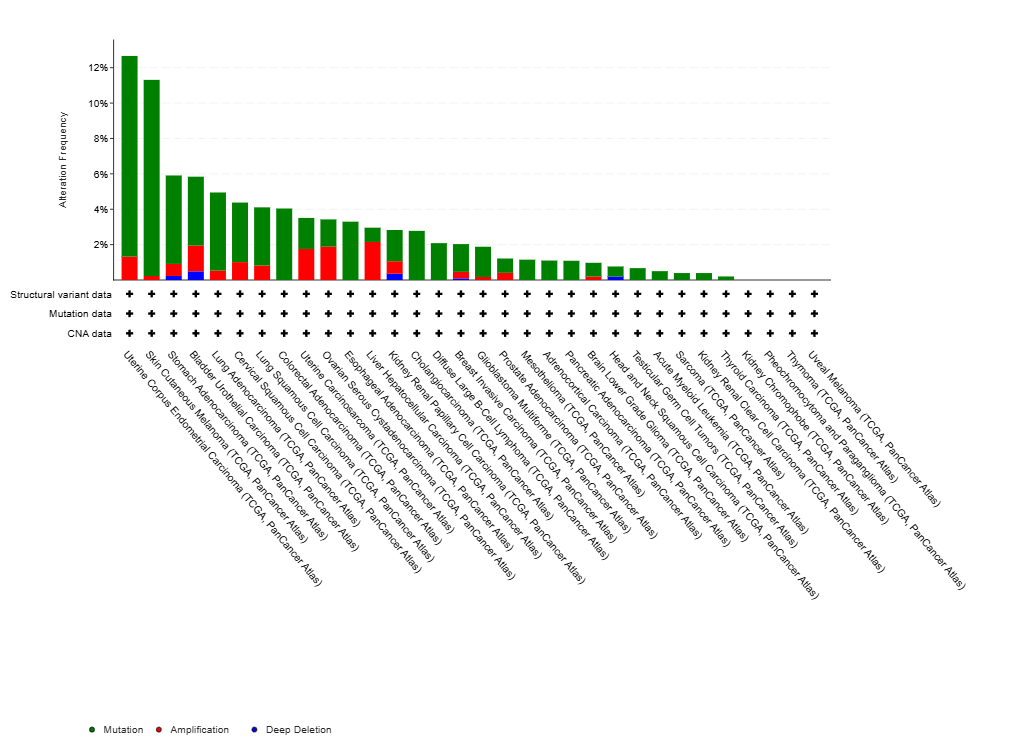

Supplement: Supplementary file 1 [file biomedicines-14-01218-s001.zip › figureS2/figureS6A.jpg]

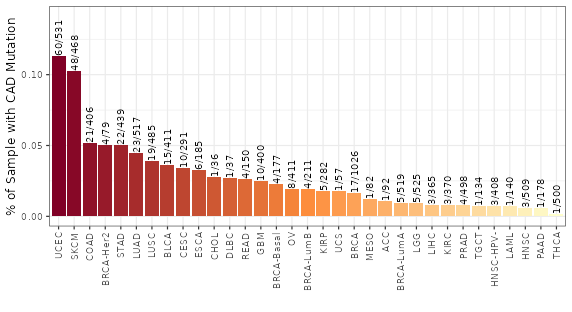

Supplement: Supplementary file 1 [file biomedicines-14-01218-s001.zip › figureS2/figureS6B.jpg]

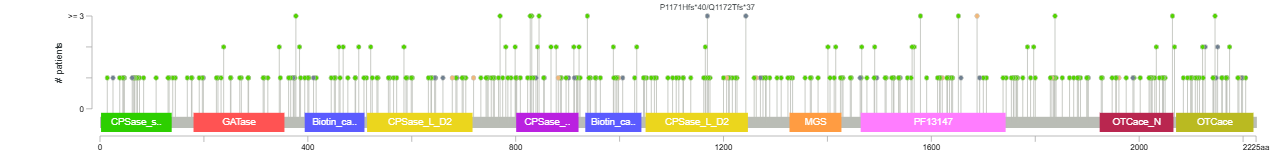

Supplement: Supplementary file 1 [file biomedicines-14-01218-s001.zip › figureS2/figureS6C.jpg]

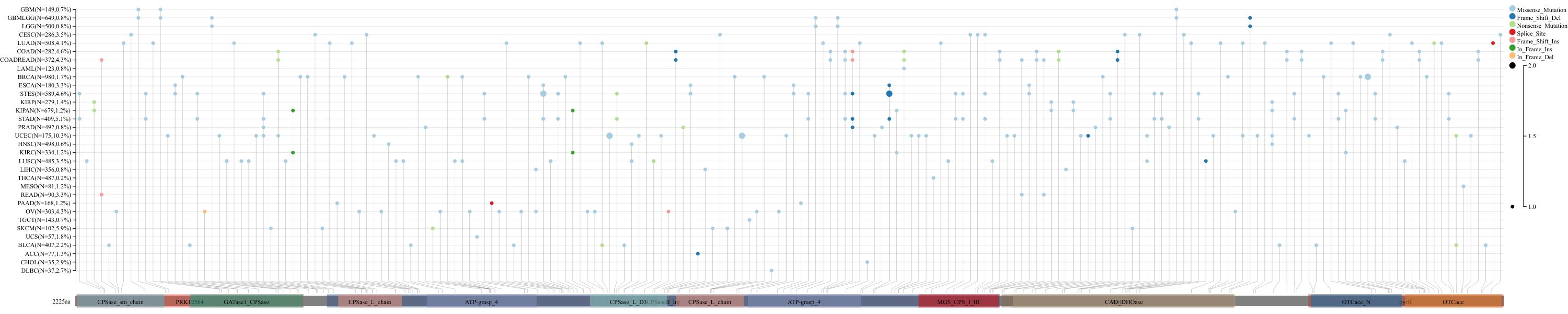

Supplement: Supplementary file 1 [file biomedicines-14-01218-s001.zip › figureS2/figureS6D.jpg]

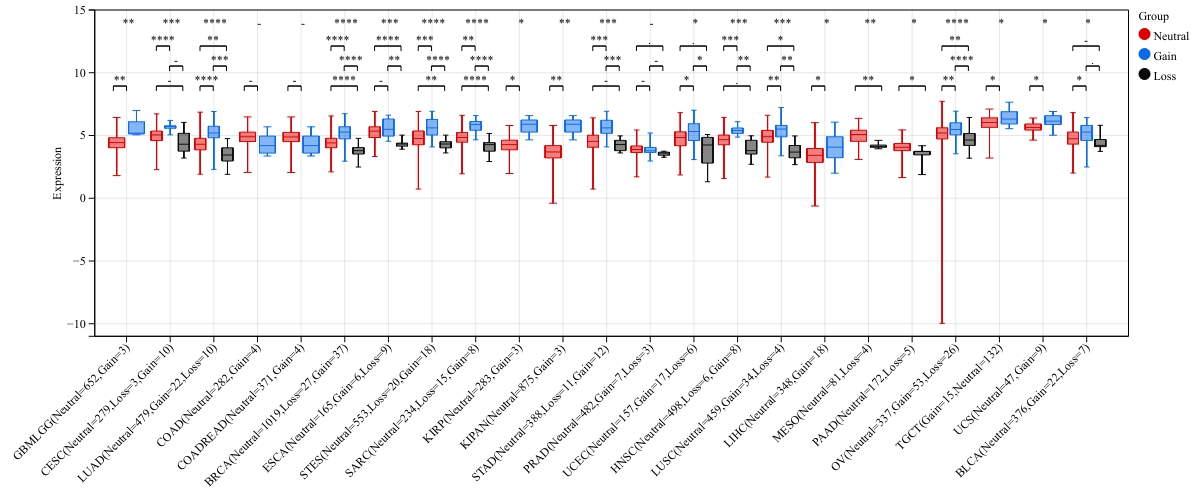

Supplement: Supplementary file 1 [file biomedicines-14-01218-s001.zip › figureS2/figureS6E.jpg]

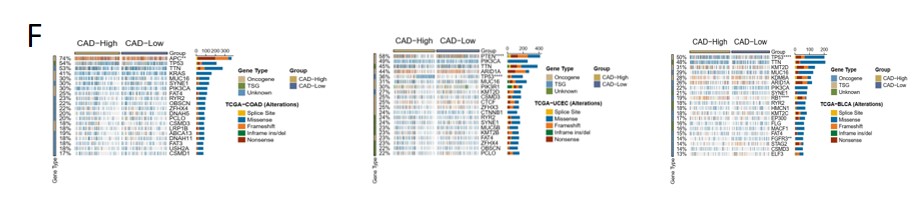

Supplement: Supplementary file 1 [file biomedicines-14-01218-s001.zip › figureS2/figureS6F.jpg]

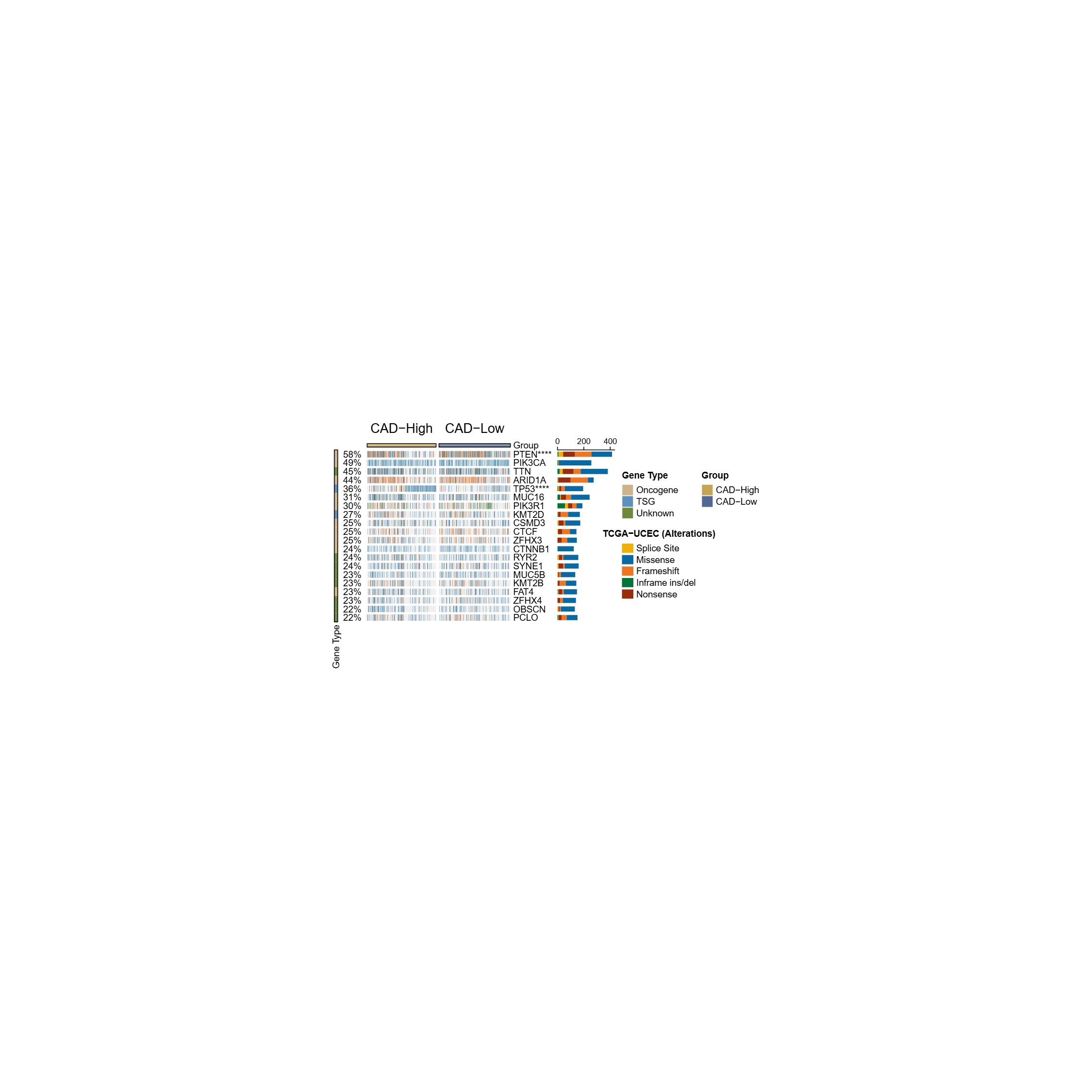

Supplement: Supplementary file 1 [file biomedicines-14-01218-s001.zip › figureS2/ucec.jpg]

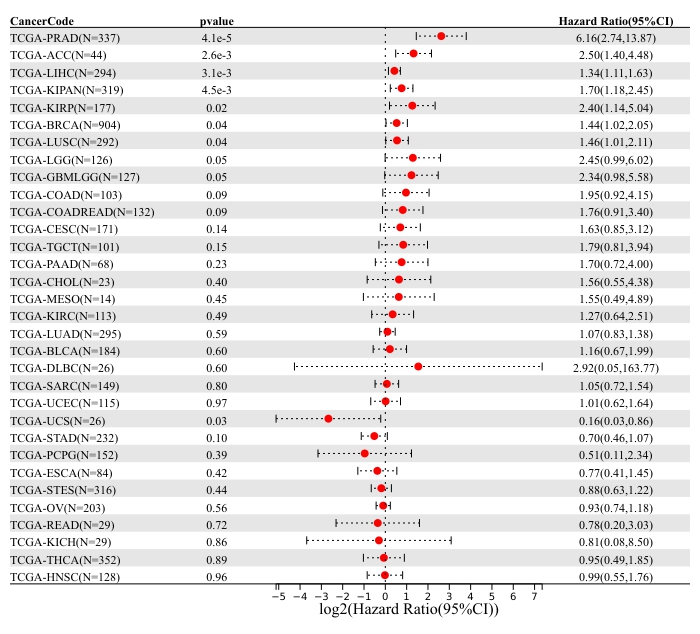

Supplement: Supplementary file 1 [file biomedicines-14-01218-s001.zip › figureS3/figureS3.jpg]

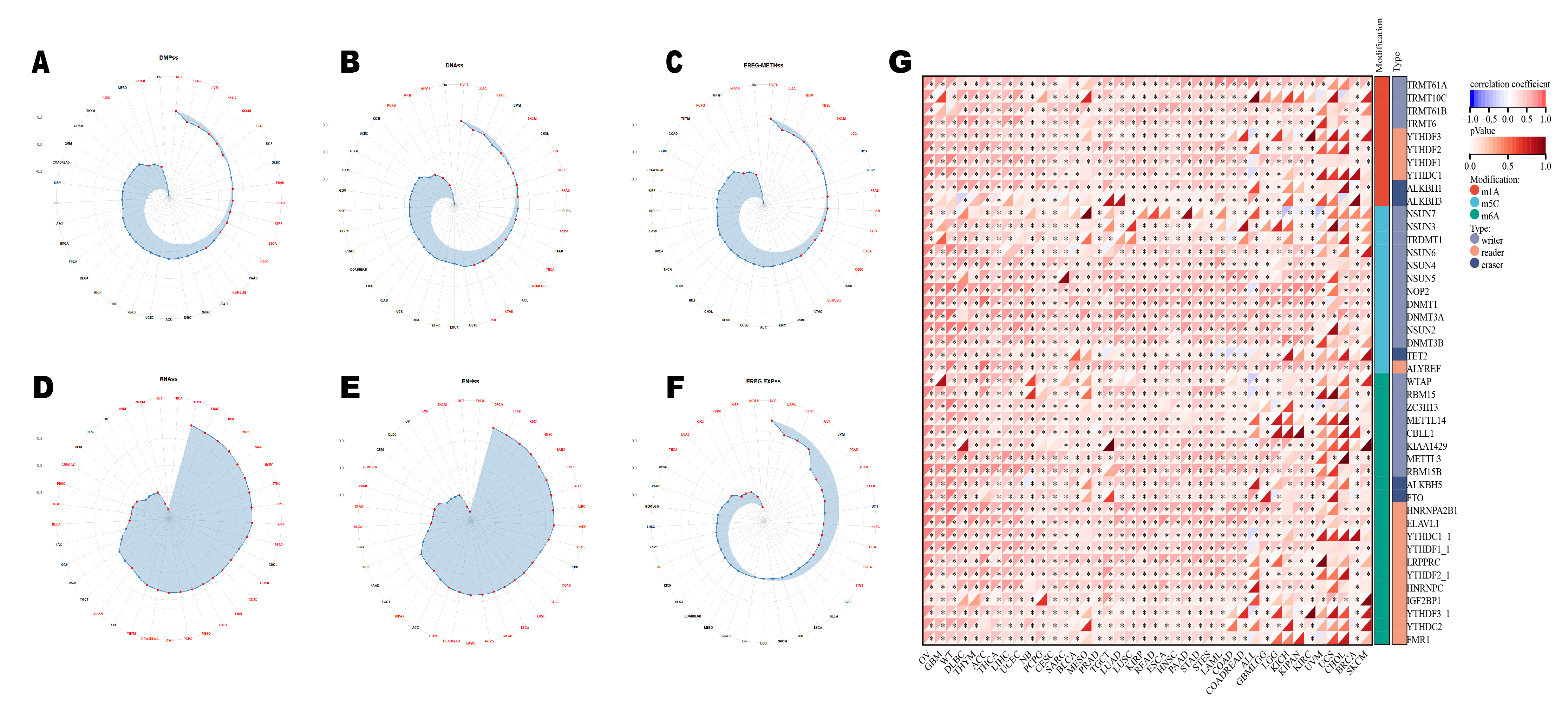

Supplement: Supplementary file 1 [file biomedicines-14-01218-s001.zip › figureS4/figureS4.jpg]

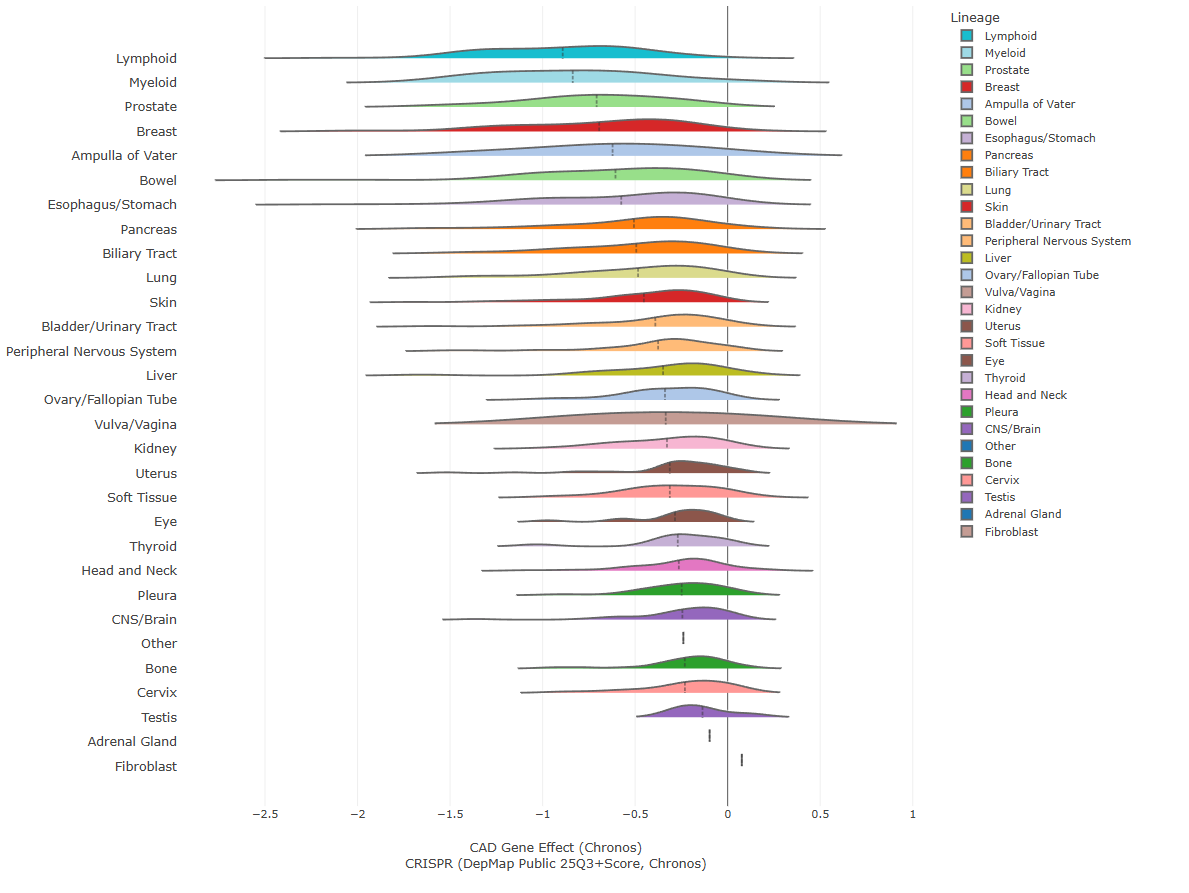

Supplement: Supplementary file 1 [file biomedicines-14-01218-s001.zip › figureS5/figureS5.jpg]
